# The CoLoMoTo Interactive Notebook: Accessible and Reproducible Computational Analyses for Qualitative Biological Networks

**DOI:** 10.3389/fphys.2018.00680

**Published:** 2018-06-19

**Authors:** Aurélien Naldi, Céline Hernandez, Nicolas Levy, Gautier Stoll, Pedro T. Monteiro, Claudine Chaouiya, Tomáš Helikar, Andrei Zinovyev, Laurence Calzone, Sarah Cohen-Boulakia, Denis Thieffry, Loïc Paulevé

**Affiliations:** ^1^Computational Systems Biology Team, Institut de Biologie de I'Ecole Normale Supérieure, Centre National de la Recherche Scientifique UMR8197, Institut National de la Santé et de la Recherche Médicale U1024, École Normale Supérieure, PSL Université, Paris, France; ^2^Laboratoire de Recherche en Informatique UMR8623, Université Paris-Sud, Centre National de la Recherche Scientifique, Université Paris-Saclay, Orsay, France; ^3^École Normale Supérieure de Lyon, Lyon, France; ^4^Université Paris Descartes/Paris V, Sorbonne Paris Cité, Paris, France; ^5^Équipe 11 Labellisée Ligue Nationale Contre le Cancer, Centre de Recherche des Cordeliers, Paris, France; ^6^Institut National de la Santé et de la Recherche Médicale, U1138, Paris, France; ^7^Université Pierre et Marie Curie, Paris, France; ^8^Metabolomics and Cell Biology Platforms, Gustave Roussy Cancer, Villejuif, France; ^9^INESC-ID/Instituto Superior Técnico, University of Lisbon, Lisbon, Portugal; ^10^Instituto Gulbenkian de Ciência, Oeiras, Portugal; ^11^Department of Biochemistry, University of Nebraska-Lincoln, Lincoln, NE, United States; ^12^Institut Curie, PSL Research University, Paris, France; ^13^Institut National de la Santé et de la Recherche Médicale, U900, Paris, France; ^14^MINES ParisTech, PSL Research University, CBIO-Centre for Computational Biology, Paris, France; ^15^Lobachevsky University, Nizhni Novgorod, Russia

**Keywords:** computational systems biology, reproducibility, model analysis, Boolean networks, Python programming language

## Abstract

Analysing models of biological networks typically relies on workflows in which different software tools with sensitive parameters are chained together, many times with additional manual steps. The accessibility and reproducibility of such workflows is challenging, as publications often overlook analysis details, and because some of these tools may be difficult to install, and/or have a steep learning curve. The CoLoMoTo Interactive Notebook provides a unified environment to edit, execute, share, and reproduce analyses of qualitative models of biological networks. This framework combines the power of different technologies to ensure repeatability and to reduce users' learning curve of these technologies. The framework is distributed as a Docker image with the tools ready to be run without any installation step besides Docker, and is available on Linux, macOS, and Microsoft Windows. The embedded computational workflows are edited with a Jupyter web interface, enabling the inclusion of textual annotations, along with the explicit code to execute, as well as the visualization of the results. The resulting notebook files can then be shared and re-executed in the same environment. To date, the CoLoMoTo Interactive Notebook provides access to the software tools GINsim, BioLQM, Pint, MaBoSS, and Cell Collective, for the modeling and analysis of Boolean and multi-valued networks. More tools will be included in the future. We developed a Python interface for each of these tools to offer a seamless integration in the Jupyter web interface and ease the chaining of complementary analyses.

## 1. Introduction

Recently, the scientific community has been increasingly concerned about difficulties in reproducing already published results. In the context of preclinical studies, observed difficulties to reproduce important findings have raised controversy (see e.g., Richter et al., 2010; Begley and Ellis, 2012; Smith and Houghton, 2013; Errington et al., 2014; and Begley and Ioannidis, 2015 for a review on this topic). Although not invalidating the findings, these observations have shaken the community. In 2016, a Nature survey pointed to the multi-factorial origin of this “reproducibility crisis” (Baker, 2016). Factors related to computational analyses were highlighted, in particular the unavailability of code and methods, along with the technical expertise required to reproduce the computations. The scientific community is progressively addressing this problem. Prestigious conferences (such as two major conferences from the database community, namely, VLDB^1^ and SIGMOD^2^) and journals (such as PNAS, Biostatistics (Peng, 2009), Nature (Santori, 2016), and Science (Yaffe, 2015), to name only a few) now encourage or even require published results to be accompanied by all the information necessary to reproduce them.

While the reproducibility challenges have first been observed in domains where deluge of data were quickly becoming available (e.g., Next Generation Sequencing data analyses), the problem is now present in many (if not all) communities where computational analyses and simulations are performed. In particular, the Systems Biology community is facing a proliferation of approaches to perform a large variety of tasks, including the development of dynamical models, complex simulations, and multiple comparisons between varying conditions of model variants. Consequently, reproducing results from systems biology studies becomes increasingly difficult. Furthermore, although the combination of different tools would provide various new scientific opportunities, this is currently hindered by technical issues.

Several initiatives have been launched by the community to address reproducibility issues for computational modeling of biochemical networks. These include guidelines for model annotations (MIRIAM, Le Novère et al., 2005) and simulation descriptions (MIASE, Waltemath et al., 2011a), as well as standards for model exchange (SBML, Hucka et al., 2003) and simulation parametrizations (SED-ML, Waltemath et al., 2011b). This collective effort is coordinated by the *COmputational Modeling in BIology NEtwork* (COMBINE^3^).

The Consortium for Logical Models and Tools (CoLoMoTo^4^) has been organized to bring together computational modeling researchers and address the aforementioned reproducibility and reusability issues within the sub-domain of logical models and software tools (Naldi et al., 2015). As a first outcome to foster model exchange and software interoperability, the SBML L3 package qual was developed (Chaouiya et al., 2013, 2015). In this manuscript, we report the next phase of the CoLoMoTo efforts in the area of reproducibility in computational systems biology: The CoLoMoTo Interactive Notebook, which provides an easy-to-use environment to edit, execute, share, and reproduce analyses of qualitative models of biological networks by seamlessly integrating various logical modeling software tools.

The teams involved in CoLoMoTo, gathering around 50 researchers within 20 groups and laboratories, have produced various software tools for the qualitative modeling and analysis of biological networks. They are also involved in the development of novel computational methods and models. This method article presents a collective effort to provide the community with a reproducibility-oriented framework combining software tools related to logical modeling. This framework combines the power of different approaches to ensure repeatability and to reduce the requirement of technical knowledge from users. The provided Docker image facilitates the stability of a contained environment needed for repeatable computational modeling and analyses. The framework includes a set of pre-installed tools from the CoLoMoTo community. On the other hand, specific binding and interfaces integrated in a Jupyter environment reduce the learning curve and improve accessibility. The use of this framework is demonstrated by a case study in a companion protocol article, which consists in a thoroughly annotated Jupiter notebook (Levy et al., 2018)^5^.

The method article is structured as follows. Section 2 provides a brief introduction to qualitative models of biological networks and to their analyses. Section 3 describes the main components (Docker image, Python programming interface, Jupyter interactive web interface) of our framework to facilitate the access to CoLoMoTo software tools, a prime prerequisite for the reproducibility of the computational analyses. Section 4 illustrates how our framework can address several challenges related to the reproducibility of computational analyses, ranging from the repeat of a sequence of analyses in the exact same software environment, to the use of alternate methods to reproduce a result. Finally, section 5 provides an introductory guide on how to use the new framework, and section 6 discusses possible extensions.

## 2. Background on qualitative dynamical models and their computational analysis

Since the pioneering work of Kauffman (1969), Thomas (1973), and others, logical (e.g., Boolean) models have emerged as a framework of choice to model complex biological networks, focusing for example on the roles of transcriptional regulatory circuits in cell differentiation and development, of signaling pathways in cell fate decisions, etc. (for a review, see e.g., Abou-Jaoudé et al., 2016).

### 2.1. Qualitative modeling

The definition of a qualitative logical model, such as a *Boolean model* usually relies first on the delineation of a *regulatory graph*, where each *node* denotes a regulatory component (e.g., a protein or a gene), while (positive or negative) *arcs* represent interactions (activation or inhibition) between their source and target nodes. Each node is modeled as a discrete variable, having a finite number of possible values, typically Boolean, i.e., only two values, 0 or 1, denoting e.g., protein absence/inactivity or presence/activity. A *Boolean function* or *rule* is then defined for each node to specify how its value may change depending on the values of its regulators.

The *state* of a network is modeled as a vector encompassing the (Boolean or multi-valued) values of all the nodes of the regulatory graph, with a prescribed ordering. The state of the network can be updated according to the logical functions defined for each node, triggering a *transition* toward a successor state. When at a given state, several nodes are called for an update, different updating modes can be considered. The *synchronous updating mode* updates all nodes simultaneously, thus leading to a unique successor state. Hence, the dynamical behavior is fully deterministic. In contrast, the *asynchronous updating mode* updates only one node, choosen non-deterministically, thus leading to different possible successor states. Several variants and extensions of these updating modes have been defined, for instance assigning pre-determined priorities or assigning probabilities to node updates, or considering simultaneous updates of sub-groups of nodes.

### 2.2. Dynamical analysis

The dynamical behavior of the model can be represented as a *state transition graph*, where vertices correspond to different states of the network, and directed edges represent *transitions* between states, following a selected updating mode. *Dynamical analyses* consist then in characterizing different properties of this state transition graph.

*Attractors* are one of the most prominent features studied in Boolean and multi-valued networks. Attractors model the asymptotic behaviors of the system, and correspond to the *terminal strongly connected components* of the state transition graph. Attractors can be of different nature, either reduced to a single *stable state* (or *fixed point*), from which no transition is possible, or *cyclic* sequences of states, modeling sustained oscillations. From a biological point of view, computing attractors is generally particularly relevant. The presence of multiple attractors can represent alternative cell fates (such as cell differentiation states), while cyclic attractors further represent periodic behaviors (such as cell cycle or circadian rhythms). The computation of attractors is addressed by different software tools, such as bioLQM (Naldi, in review^6^), GINsim (Naldi et al., 2018), Pint (Paulevé, 2017), BoolSim (Garg et al., 2008), BooleanNet (Albert et al., 2008), pyBoolNet (Klarner et al., 2017), and BoolNet (Müssel et al., 2010).

*Simulations* allow capturing the states *reachable* from a given (set of) *initial state(s)*. They can consist of random walks in the complete state transition graph, take into account updating priority schemes to distinguish fast versus slow processes and thereby obtain a simpler state transition graph (Fauré et al., 2006), as implemented in the software tool bioLQM, or rely on user-defined transition probabilities and timing, as implemented into the software tools MaBoSS (Stoll et al., 2012, 2017) and CellCollective (Helikar et al., 2012; Todd and Helikar, 2012).

*Model checking* techniques developed for software verification in computer science allow verifying formally dynamical properties on state transition graphs and are regularly employed for analysing biological systems (Batt et al., 2005; Abou-Jaoudé et al., 2015; Bartocci and Lió, 2016; Traynard et al., 2016). The properties are specified using so-called *temporal logics*, which enable the formulation of queries regarding asymptotic or transient dynamical properties, taking into account all the state transitions of the model. The accordance of a Boolean/multi-valued model with such properties is verified using a general purpose model checker such as NuSMV (Cimatti et al., 2002) to which GINsim and Pint provide access.

It is worth noticing that the number of states of a Boolean or multi-valued network grows exponentially with the number of nodes. The above mentioned methods typically suffer from this complexity, and hence face limitations regarding network size (currently, this limit is of the order of fifty to a hundred of nodes, depending on the analysis and the complexity of the dynamics). Nevertheless, different approaches enable the analysis of large scale qualitative networks by means of structural analyses, model reductions or abstractions. The CoLoMoTo Interactive Notebook provides access to methods for *model reductions*, such as by Naldi et al. (2011), implemented in bioLQM, which preserves stable states, while cyclic attractors and reachability can be affected in predictable ways, or by using *formal approximations* of the dynamical behavior, as implemented in Pint, which allow tackling networks with several thousands of nodes (Paulevé, 2017, in press). Other approaches include, for instance, Petri net model reduction for trajectories in signaling pathways (Talcott and Dill, 2006), subnetwork analysis (Siebert, 2009), computational algebra (Veliz-Cuba et al., 2014), and motif-based abstractions for attractors (Gan and Albert, 2018).

**Figure 1:**
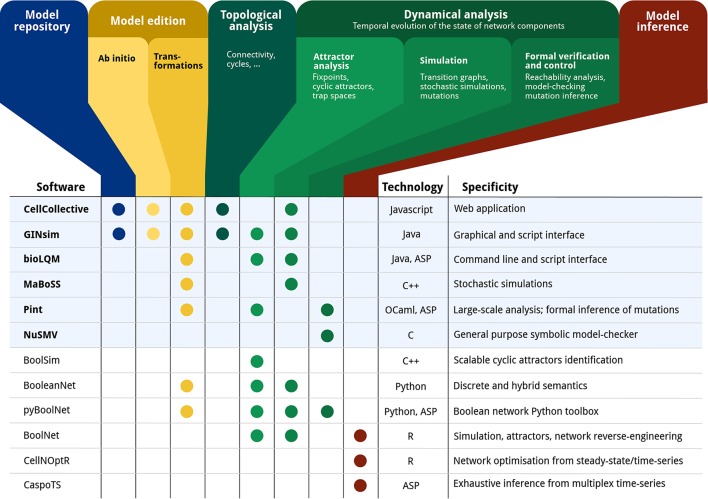
Feature matrix and characteristics of a range of software tools devoted to the qualitative modeling and analysis of biological networks: cellcollective (Helikar et al., 2012); ginsim (Naldi et al., 2018); biolqm (Naldi, in review^6^); maboss (Stoll et al., 2017); pint (Paulevé, 2017); nusmv (Cimatti et al., 2002); boolsim (Garg et al., 2008); booleannet (Albert et al., 2008); pyboolnet (Klarner et al., 2017); boolnet (Müssel et al., 2010); cellnopt (Terfve et al., 2012); caspots (Ostrowski et al., 2016). The current CoLoMoTo Docker (2018-03-31) ships the software indicated with a bold font and a light blue background. “Model repository” refers to searchable databases of models; “Model edition” refers to the features related to creating and modifying a qualitative model, where “*ab initio*” refers to the interactive model building from scratch, and “transformations” refers to operations such as mutations, Booleanization, model reduction, etc. “Topological analysis” refers to the extraction of features from the regulatory graph, such as the different feedback cycles, graph theory measures, etc. “Dynamical analysis” refers to properties related to the state transition graph of Boolean/multi-valued networks, where “Attractor analysis” refers to the identification of stable states, cyclic attractors, and related features; “Simulation” refers to the sampling of trajectories within the state transition graph, possibly parameterized with stochastic rates and mutations; “Formal verification and control” refers to exhaustive analyses for assessing strictly temporal properties, such as reachability, and deducing mutations for controlling the system. Finally, “Model inference” refers to the derivation of Boolean/multi-valued network which are compatible with given properties and observation data.

Figure 1 gives an overview of a range of software tools for the analysis of qualitative models, specifying their main features along with the main underlying technologies.

## 3. Accessibility of CoLoMoTo software tools

The CoLoMoTo Interactive Notebook aims at offering a unified environment for accessing a range of complementary software tools for the analysis of qualitative models of biological networks. To achieve such a goal, our framework relies on three complementary technologies.

First, we use the Docker system to provide images of pre-installed selected CoLoMoTo software tools, thus reducing significantly the burden of installing individually each software tool. The software installed within Docker images can be executed on GNU/Linux, macOS, and Microsoft Windows, and can be accessed by standard workflow systems, such as SnakeMake (Köster and Rahmann, 2012).

Then, we developed a collection of Python modules to provide a unified interface to the features of the selected software tools. The Python modules allow to parameterize and execute the different analyses, and fetch their results, which can then be further processed, including by a different tool through its respective Python module. This uniform Python interface is particularly relevant in the Jupyter web interface (Ragan-Kelley et al., 2014), where it allows editing executable notebooks on qualitative biological networks by seamlessly combining different software tools.

### 3.1. The CoLoMoTo docker image

Overall, we witness a growing ecosystem of software tools based on different technologies and offering a wide range of complementary features. Noteworthy, these tools typically rely on tailored formalisms and settings, which enable specific methods but at the same time affect the results. One obvious example is the consideration of a specific updating mode, as synchronous and asynchronous dynamics may differ extensively. Furthermore, to address increasingly large networks, many tools rely on advanced data-structures and resolution methods, which are implemented in dedicated software libraries. The distribution of these tools then become challenging, as they rely on numerous dependencies, often difficult to install or available only for a specific operating systems (most of the time GNU/Linux).

The Docker container technology allows to circumvent such distribution issues by providing a mean to supply pre-installed and fully configured software environments in so-called *Docker images*. On GNU/Linux, the execution of a Docker image consists mainly in executing the software in an isolated environment, requiring no operating system virtualization. Therefore, the overhead of using Docker on GNU/Linux is close to zero. A Docker image can also be executed on macOS or Microsoft Windows without any modification. On these operating systems, Docker relies on virtualization technologies, which are relatatively lightweight and result in limited performance loss on recent hardware.

The current CoLoMoTo Docker image colomoto/colomoto-docker:2018-03-31 contains the following pre-installed software for the logical modeling and analsyis of biological networks: GINsim (Naldi et al., 2018), bioLQM (Naldi, in review)^6^, CellCollective (Helikar et al., 2012), MaBoSS (Stoll et al., 2017), Pint (Paulevé, 2017), and NuSMV (Cimatti et al., 2002). The CoLoMoTo Docker image then provides access to these tools without requiring any installation step beside installing Docker^7^. For instance, the Docker image can be used in association with a workflow manager to chain and run a series of software functionalities. Supplementary File “*SnakeMake*” provides an example of SnakeMake workflow relying on GINsim and NuSMV.

An important challenge is the maintenance and extendibility of such Docker images to reduce the complexity of upgrading or adding software tools with their respective dependencies. To that aim, we require that each software tool is independently packaged for GNU/Linux using the *Conda package manager*^8^. We then rely on the dependency management system of Conda to ensure that the correct pre-requisites are installed in the Docker image^9^. A beneficial side effect of this technical choice is that the aforementioned software tools can be installed on GNU/Linux platforms using Conda, without using Docker.

### 3.2. A unified interface for calling and chaining tools with python

The software tools considered for the CoLoMoTo Docker image present different interfaces: CellCollective is a web application, GINsim has a graphical user interface along with a scripting interface, bioLQM has a command line and a scripting interface, Pint has a command line and a Python interface, MaBoSS has a command line interface. GINsim, CellCollective and bioLQM support the SBML-qual format, while bioLQM provides the conversion of a standard SBML-qual model into Pint or MaBoSS model formats, thereby enabling the exchange of models between all these tools.

The recourse to different interfaces complicates the design of a model analysis combining multiple tools. To address this issue, we have developed a Python interface for each of the tools embedded in the CoLoMoTo Docker image, which greatly ease the execution of different tool functionalities, fetch the results, and use these as input for other executions.

Each tool comes with a dedicated Python module, providing a set of functions to invoke the underlying software tool appropriately. Therefore, from a single Python shell, one can invoke and chain analyses performed by different tools. This can be seen as an improved command line interface, greatly enhanced by the use of intermediate Python objects. Such an approach also promotes the use of standard Python data-structures to store objects such as model states or graphs, which can then be processed by common Python libraries, e.g., Pandas^10^ or NetworkX^11^.

Hereafter, we give an overview of the resulting Python programming interface, focusing on the general model input mechanism and the main features implemented for each of the software tools.

#### 3.2.1. Model input and tool conversions

Despite their very different features, all the tools considered here take as input a logical model, in an adequate format. All the related Python modules provide a load function, which takes as input the location of the model, being a local file, for instance:


m = biolqm.load(“path/to/localfile.sbml”)


a web link to a file, as obtained on GINsim repository^12^ for instance:



m = biolqm.load(“http://ginsim.org/sites/default/files/Traynard_Boolean_MamCC_Apr2016.sbml”)


or a web link to the model on CellCollective, for instance:


m = biolqm.load(“https://cellcollective.org/#5128/lac-operon”)


In each case, the returned object (identified by m in the above examples) is a Python object representing the loaded model and defined specifically for the corresponding tool (Python module). Table 1 lists the supported input format for each software tool.

**Table 1:** Model input formats for the software tools included in the CoLoMoTo Docker image.

**Software tool**	**Supported input formats**
bioLQM	SBML-qual (.sbml), raw logical functions, truth table
GINsim	GINML (.ginml, .zginml)
Pint	Automata network (.an)
MaBoSS	Dedicated network/configuration files (.bnd/.cfg)
NuSMV	SMV file (.smv)

**Table 2:** List of notebook files in supplemental data "Notebooks" (Data Sheet 2) demonstrating some features of the CoLoMoTo Interactive Notebook.

**Notebook file name**	**Software tools involved**
demo-cellcollective	CellCollective, bioLQM
demo-ginsim	GINsim, bioLQM
demo-biolqm	bioLQM
demo-interactive-fixpoints	bioLQM
demo-pint	Pint
demo-nusmv	GINsim, NuSMV
demo-maboss	MaBoSS
demo-pint+maboss	CellCollective, bioLQM, Pint, MaBoSS
demo-reproducibility-fixpoints	GINsim, bioLQM, Pint
demo-reproducibility-modelchecking	GINsim, Pint

**Figure 2:**
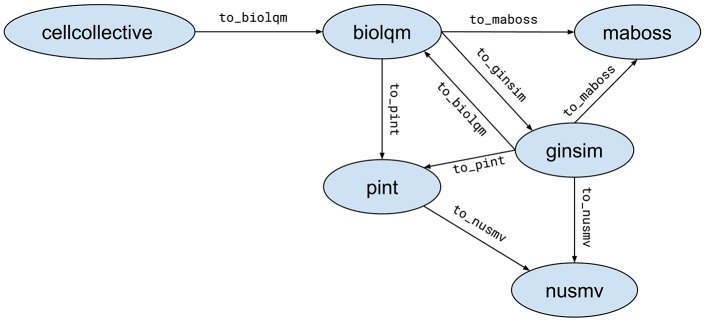
Supported model conversions between Python modules.


When possible, Python modules provide functions to convert a model for a compatible tool. These functions are of the form moduleA.to_moduleB(modelA). Figure 2 lists the currently supported model conversions. The following Python code shows an example of usage:

lrg = ginsim.load(“http://ginsim.org/sites/default/files/Traynard_Boolean_MamCC_Apr2016.sbml”)
lqm = ginsim.to\_biolqm(lrg)
an = biolqm.to\_pint(lqm)


Here, lrg is a Python object representing a GINsim model, lqm is a Python object representing a bioLQM model, and an is a Python object representing a Pint model.

#### 3.2.2. CellCollective – modeling platform, repository, and knowledge base

The cellcollective Python module allows connecting to the CellCollective (Helikar et al., 2012) web application (https://www.cellcollective.org), in order to download the models in SBML-qual format and extract network node meta-data (e.g., UnitProt identifiers) when available. The Supplementary File “*Notebooks/demo-cellcollective*”^13^ provides a brief demonstration of the Python module usage.

#### 3.2.3. GINsim – regulatory network modeling

The ginsim Python module provides direct access to the Java programming interface of GINsim (Naldi et al., 2018). GINsim is available and documented at http://www.ginsim.org. In particular, besides the export of a GINsim model into various file formats, the Python module allows to visualize the network regulatory graph, with the activation and inhibition relationships between the nodes. The visualization function (ginsim.show) optionally takes as argument a Python dictionary associating a level with each node; then, the nodes of the network are colored according to these levels. This is illustrated in the Supplementary File “*Notebooks/demo-ginsim*”^14^.

#### 3.2.4. bioLQM – qualitative model toolbox

The biolqm Python module provides direct access to the Java programming interface of bioLQM (Naldi, in review^6^). bioLQM is available and documented at http://colomoto.org/biolqm. bioLQM supports the conversion of SBML-qual files, GINML files, as well as simple textual files specifying the raw logical functions into the formats associated with the different software tools. Besides the file format features, bioLQM implements *model modifications*, such as mutations forcing the value of given nodes, iterative model reduction (see above), model reversal, the conversion of multi-valued model into Boolean ones, as well as the computation of *stable states, trap spaces*, and *simulations*. Part of these features are illustrated in the Supplementary File “*Notebooks/demo-biolqm*”^15^.

#### 3.2.5. Pint – formal predictions for controlling trajectories

The pypint Python module provides complete access to features documented at https://loicpauleve.name/pint. The software Pint is devoted to the analysis of trajectories in very large-scale asynchronous Boolean and multi-valued networks (Paulevé, 2017). Its main features include the verification of the existence of a trajectory reaching a state of interest (*reachability*), the identification of common points between *all* the trajectories leading to a state of interest (*cut sets*), and the *formal prediction of mutations* preventing the existence of any trajectory to the given state. These features are illustrated in the Supplementary File “*Notebooks/demo-pint*”^16^.

#### 3.2.6. NuSMV – model verification

The nusmv Python module provides a simple interface to the NuSMV model checker for verifying LTL (trace) and CTL (computation tree) temporal logic properties (Cimatti et al., 2002) . The specification of LTL and CTL properties can be facilitated using the colomoto.temporal_logics Python module, which takes advantage of Python objects for the different logical operators and ease their combination.

Let us consider the following example using the CTL operators from the aforementioned Python module:


p1 = AG (S(a=1))
p2 = EF (p1)


Here, the variable p1 is a CTL formula specifying that the node *a* is active (S(a=1)) in all the reachable states (AG operator). The variable p2 is a CTL formula specifying that there exists a trajectory leading to a state (EF operator) from which the property p1 is verified.

In the above example, S specifies a property on a state, by giving the values of some nodes of the network. The conversion of a network model into NuSMV format depends on the tool used, sometimes introducing different variable names for the nodes of the original biological network. But this technical point is transparent for the user: the nusmv Python module will automatically translate the node names into the correct NuSMV variable names.

The Supplementary File “*Notebooks/demo-nusmv*”^17^ gives a simple example of usage of the nusmv Python module to verify properties of a GINsim model.

#### 3.2.7. MaBoSS – stochastic simulations

The maboss Python module provides an interface to MaBoSS, available at https://maboss.curie.fr, as well as basic plotting functionalities (Stoll et al., 2017). The purpose of MaBoSS is to perform stochastic simulations of a Boolean network, where the propensity of transitions (probabilistic rates) are explicitly specified. The Python module allows to fully define and parameterize a model, as well as to parse an existing MaBoSS model and modify it programmatically. The object returned after the simulations can then be used to plot the probability of node activation over time, and the proportion of states in which the simulations ended, in order to estimate the probability of reaching different attractors. The Supplementary File “*Notebooks/demo-maboss*”^18^ provides a brief tutorial to the main features of the maboss Python module.

#### 3.2.8. Advanced combinations of tools

These Python modules provide a unified interface to chain different tools and process their results. The small tutorials referenced above show simple chaining of tools, most of the time using a tool to import a model (e.g., from CellCollective or GINsim) and convert it (using bioLQM) for specific analysis by another tool. As the Python functions of the different modules rely on standard Python data-structures, such as lists and dictionaries, it is possible to easily re-use the result from a tool function as input to the function of a different tool. A simple example is provided in Supplementary File “*Notebooks/demo-ginsim*”^19^, where we use bioLQM to compute the stable states of a GINsim model, and then give one of the resulting state as input to GINsim show function to display it over the regulatory graph.

Moreover, one can use the programmatic features of Python to implement advanced algorithms for executing multiple analyses and process their results. For instance, one can program loops to iterate over a list of results of a preceding analysis from one tool to perform a subsequent analysis on each result with another tool. This is illustrated in the Supplementary File “*Notebooks/demo-pint+maboss*”^20^, where we use Pint to formally predict combinations of mutations controlling the existence of trajectories toward a specified state; then, we quantify with MaBoSS the efficiency of applying only partially the predicted combinations, by evaluating each related double-mutants. The example involves Python for loops and a function to enumerate all possible subsets provided by the standard Python library. The notebook also relies on CellCollective to fetch the model, and on bioLQM to perform the adequate model conversions.

### 3.3. CoLoMoTo jupyter interactive notebook

*Jupyter*^21^ is a software providing an interactive web interface for creating documents, called *notebooks*, mixing code, equations, and formatted texts. A notebook typically describes a full analysis workflow, combining textual explanations, the code itself, along with parameters to reproduce the results. A notebook is a single file, which can be easily modified, shared, re-executed, and visualized online. The short tutorials of the previous section provided in the Supplementary File “*Notebooks*” are actually Jupyter notebooks (files with the extension .ipynb) and can be re-executed using Jupyter.

A Jupyter notebook is made of a sequence of so-called *cells*, which can contain formatted text, including sections, links, images, tables, etc., or which can contain code in a specified programming language, typically Python. A code cell can be executed (by pressing Shift-Enter) and the value returned by the code is displayed below the cell. The display format is selected according to the type of the returned value (image, graph, list, table, …) to offer an adequate visualization.

Having a unified Python interface to invoke the CoLoMoTo software tools, one can directly create Jupyter notebooks for the analysis of qualitative biological networks using these tools, as shown in Supplementary File “*Notebooks*” and in the companion publication providing a complete model analysis workflow (Levy et al., 2018).

**Figure 3:**
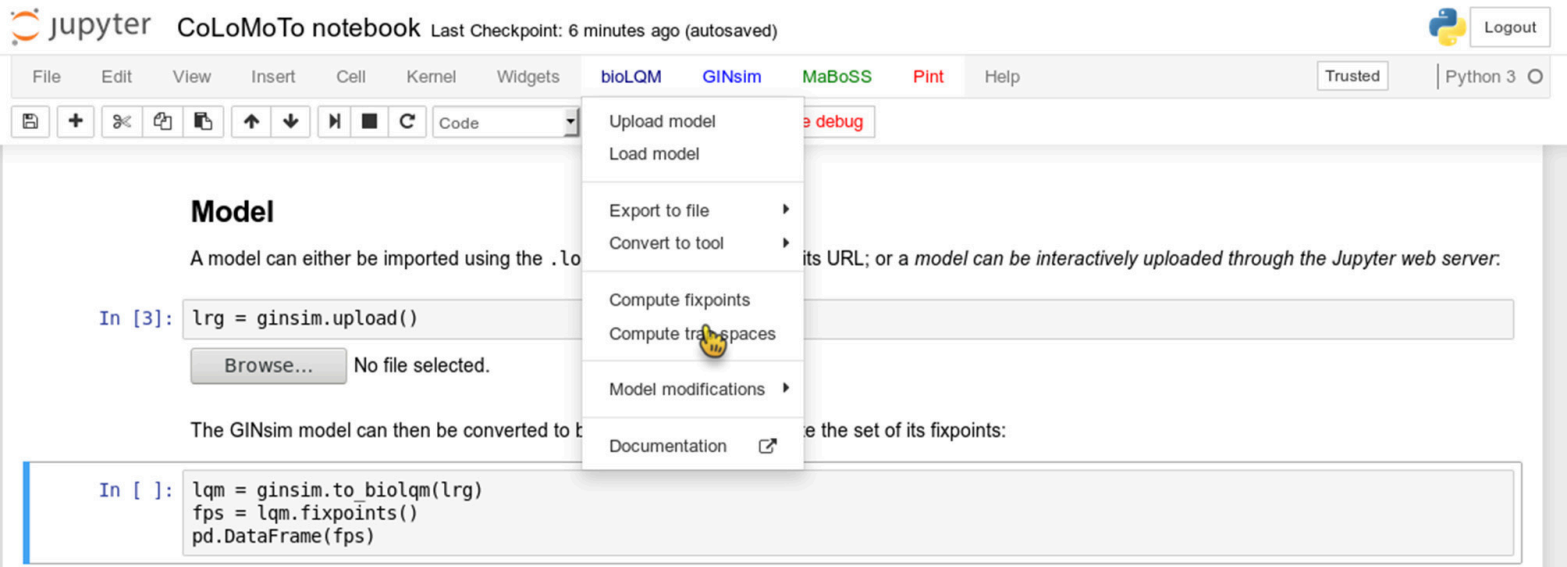
Screenshot during a Jupyter notebook edition showing the menu of the bioLQM tool.

We added several features in the CoLoMoTo Python modules to increase interactivity and improve the user experience for editing Jupyter notebooks. First, menus provide pre-defined Python code for accessing to the main features of the tools. Figure 3 shows a screenshot during the edition of a Jupyter notebook with its graphical interface. Next, we added the possibility to interactively upload a model file. This feature is particularly useful when used in combination with Docker, or on a remote server with no direct access to the user file system. Finally, some Python modules, in particular the maboss Python module, provide JavaScript widgets to generate Python code interactively.

The Jupyter notebook server is included in the CoLoMoTo Docker image (see the Discussion section for a quick usage guide), while a public demonstration web instance is available at http://tmpnb.colomoto.org.

## 4. Reproducibility of computational analyses

### 4.1. From repeatability to reproducibility

The literature provides a range of definitions for the reproducibility of *in silico* experiments by analogy to wet lab experiments (Drummond, 2009; Freire et al., 2012; Stodden et al., 2013; Freire et al., 2016; Goodman et al., 2016; Lewis et al., 2016; Cohen-Boulakia et al., 2017). Four *levels* of reproducibility are commonly distinguished.

An *in silico* experiment is said to be *repeated* when it is performed using the same computational set-up as the original experiment. The major goal of the *repeat* task is to check whether the initial experimental result was correct and can be obtained again. The difficulty lies in recording as much information as possible to repeat the experiment so that the same conclusion can be drawn. Interestingly, Freire et al. (2012) discusses the granularity at which information (experiments, data sets, parameters, environment) should or could be recorded, and underlines the fact that the key point is to determine the right balance between the effort required to record information and the capability of obtaining identical results.

An *in silico* experiment is said to be *replicated* when it is performed in a new setting and computational environment, although similar to the original ones. When it can be successfully replicated, a result has a high level of robustness: it remained valid when using a similar (although different) protocol. A continuum of situations can be considered between repeated and replicated experiments.

A result is then defined as *reproduced*, in the broadest possible sense of the term, by denoting the situation where an experiment is performed within a different environment, with the aim to validate the same scientific hypothesis. In other words, what matters here is the conclusion obtained and not the methodology considered reaching it. Completely different approaches can be designed, different data sets can be used, as long as the experiments support the same scientific conclusion. A reproducible result is thus a high-quality result, confirmed in various ways.

A last important concept related to reproducibility is that of *reuse*, which denotes the case where a *different* experiment is performed, with similarities with an original experiment. A specific kind of reuse occurs when a single experiment is reused in a new context (and thus adapted to new needs), the experiment is then said to be *repurposed*.

It is worth noticing that *repeating* and *replicating* may appear to be technical challenges compared to *reproducing* and *reusing*, which are the most important scientific objectives. However, before investigating alternative ways of obtaining a result (to reach reproducibility), or before reusing a given methodology in a new context (to reach reuse), the original experiment has to be carefully tested, especially by reviewers or any peer, demonstrating its ability to be at least repeated and hopefully replicated (Freire et al., 2012; Stodden et al., 2014).

### 4.2. Repeat analysis in the same software environment

Ensuring that a sequence of computational analyses can be repeated by other scientists several months or years after its publication is difficult. Indeed, besides software availability, the version of the tools can be crucial: a new version of a tool can change the default parameters, and even some features, so that the published instructions become obsolete. Whereas a Docker image addresses efficiently the issue of making software available, providing a safe way for repeating a notebook content years after its creation requires additional technical procedures.

First, CoLoMoTo Docker images are constructed by specifying explicitly the version of each software. Furthermore, an automatic validation procedure is performed by checking that a set of notebooks still execute without error. Once validated, the Docker image is then tagged with a time-stamp, typically the date of the image validation (of the form YYYY-MM-DD, e.g., 2018-03-31). These tagged images are then stored in the public Docker image registry, and can be retrieved any time later. The list of existing tags of colomoto/colomoto-docker Docker images can be viewed at https://hub.docker.com/r/colomoto/colomoto-docker/tags/.

When sharing a notebook, and notably when attaching it to a publication, it is highly recommended to specify the time-stamp of the Docker image in which the notebook has been executed. Then, by downloading the image with this specific tag, other users are ensured to repeat the execution in the exact same software environment. To help following this recommendation, we took two technical decisions. First, we do not use the default non-persistent tag for Docker images (*latest*). It means that the user has always to specify explicitly the time-stamp of the CoLoMoTo Docker image. To remove the burden of actively checking the list of existing time-stamps, we provide a script which, by default, fetches the most recent Docker image (see section 5). Second, when loading a CoLoMoTo-related Python module within a Docker container, a textual message indicating the time-stamp of the Docker image is displayed. Therefore, when created within a CoLoMoTo Docker image, notebooks always contain the required information to repeat their execution.

Because a Jupyter notebook is a single file containing everything to execute it, one can easily check if it can be *replicated* in a different software environment, e.g., using a more recent CoLoMoTo Docker image. Moreover, a notebook can be easily *repurposed* by modifying some arguments of the Python function calls, for instance changing the input model or analysis parameters. One can even define interactive notebooks describing a common model analysis, so that the user only needs to provide the input model and execute the Jupyter code cells, as shown in the Supplementary File “*Notebooks/demo-interactive-fixpoints*”^22^ for the computation and visualization of the stable states of a bioLQM model.

### 4.3. Reproduce analysis with a different method

Reproducing the same analysis with two different methods is a good mean to increase confidence in the results, as it reduces the chance of software misuse or that the results are affected by a software bug.

The subset of software tools selected for this first CoLoMoTo Docker image presentation already provides redundant implementations of equivalent model analyses, in particular for the identification of stable states and for the verification of temporal properties with NuSMV. To help switch between two tools for performing the same task, we harmonized the usage of Python module functions to ensure that the same functions with the same arguments generate equivalent results with different tools.

#### 4.3.1. Stable states

There exists several methods to compute the full set of stable states (or fixed points) of a logical model, relying on different data-structures and different algorithms. The software bioLQM implements the computation of stable states for Boolean and multi-valued logical models using a Java implementation of decision diagrams. In contrast, the software Pint implements the computation of stable states of Automata networks (a generalization of logical networks) using Boolean satisfaction constraints. As bioLQM provides a conversion of Boolean/multi-valued network into equivalent Automata networks, it is possible to compute the stable states of a model with both software tools.

Both biolqm and pypint Python modules provide a fixpoint function taking as input the model instance of the corresponding tool and returning a list of Python dictionaries describing the stable states. Provided lqm is a bioLQM model, the following Python code compute its stable states with both tools:


fps_biolqm = biolqm.fixpoints(lqm)
fps_pint = pypint.fixpoints(biolqm.to_pint(lqm))


The Supplementary File “*Notebooks/demo-reproducibility-fixpoints*”^23^ shows a complete example of reproduction of stable state computation using BioLQM and Pint.

#### 4.3.2. Temporal property verification (model-checking)

Both GINsim and Pint allow to export their respective model into NuSMV format, where temporal properties can be specified using LTL or CTL (see section 2.2). However, the generated NuSMV models have different features as the input formalisms of these tools rely on different paradigms: the specification is centered on logical rules in the case of Boolean/multi-valued networks in GINsim, and on transitions (à la Petri nets) in the case of Automata networks in Pint. Nevertheless, in the appropriate settings, the verification of an equivalent CTL or LTL property should give the same result. Hence, the functions ginsim.to_nusmv and pypint.to_nusmv are implemented in such ways that, when using their default options, the resulting NuSMV models, albeit different, should produce identical results for identical temporal logic properties. Note, however, that each tool provides specific options for the NuSMV export, which can lead to incomparable results.

The following Python code uses operators defined in the Python module colomoto.temporal_logics to specify a property p, meaning that from any state, there always exists a trajectory leading to a cyclic attractor where the level of node *a* can always oscillate. Then, assuming lrg is a GINsim model, the code uses GINsim and Pint conversions to NuSMV to perform model verification.


p = EF (AG (EF (S(a=0)) & EF (S(a=1))))
  
nusmv_ginsim = ginsim.to_nusmv(lrg)
nusmv_ginsim.add_ctl(p)
nusmv_ginsim.verify()
  
nusmv_pint = pypint.to_nusmv(ginsim.to_pint(lrg))
nusmv_pint.add_ctl(p)
nusmv_pint.verify()


Note that the Python object p represents the CTL property to be tested, whatever the origin of the model (GINsim or Pint).

The Supplementary File “*Notebooks/demo-reproducibility-modelchecking*”^24^ provides a more detailed example of the reproduction of model-checking results using GINsim and Pint.

## 5. Quick-usage guide

On GNU/Linux, macOS, or Microsoft Windows, provided that Docker and Python are installed, a helper script to run the CoLoMoTo Docker image and the embedded Jupyter notebook can be installed and upgraded from a terminal using the following command^25^:


pip install -U colomoto-docker


The Docker image and the Jupyter notebook interface can be started by executing the following command in a terminal^26^:


colomoto-docker


Without any argument, the command will use the most recent CoLoMoTo Docker image. To use the image with a specific tag, append the -V option (e.g., colomoto-docker -V 2018-03-31).

The execution of this command will open a web page with the Jupyter notebook interface, enabling loading and execution of notebooks. A new notebook can be created by using the “New/Python3” menu. In this environment, the user has access to all CoLoMoTo Python modules. A code cell is executed by typing “Shift+Enter.” The menu and tool bar allow quick access to the main Jupyter functionalities.

Warning: by default, the files within the Docker container are isolated from the running host computer, and are deleted when stopping the container. To have access to the files of the current directory of the host computer, the option --bind can be used:


colomoto-docker --bind .


The container can later be stopped by pressing Ctrl+C keys in the terminal. See colomoto-docker --help for other options. Additional documentation for running the CoLoMoto Docker image can be found at http://colomoto.org/notebook.

## 6. Discussion

### 6.1. Academic use cases

The prime aim of the CoLoMoTo Interactive Notebook is to foster the production of accessible and reproducible computational analysis of biological models, with a focus on qualitative models, including Boolean and multi-valued networks. As demonstrated in the Supplementary File “*SnakeMake*”, the CoLoMoTo Docker image can also be used in standard workflow systems, such as SnakeMake, to lighten the burden of installing the different software tools and make them accessible on different operating systems.

A notebook issued from the CoLoMoTo Docker image gives some guarantees of repeatability, as it contains references to the persistent Docker image to re-execute code in the same software environment. Therefore, the notebook file (with .ipynb extension) can be distributed as a Supplementary File of the related scientific article, along with instruction to run the Docker image. The Jupyter interface further allows to export the notebook in a static HTML file, which could also be joined as a Supplementary File to provide a quick visualization. A notebook can also be distributed independently, for instance by publishing it on *Gist*^27^ or *myExperiment*^28^ (Goble et al., 2010), to follow download and potential updates. For instance, the tutorial notebook presented by Levy et al. (2018) is hosted at https://gist.github.com/pauleve/a86717b0ae8750440dd589f778db428f. Services like Zenodo^29^ further provide persistent DOI links to notebook files.

The CoLoMoTo Interactive Notebook is also relevant for teaching purposes. With Jupyter, students can straightforwardly execute, modify, and extend a template notebook to learn methods for analysing models of biological networks. Docker is a standard technology often supported by local cloud infrastructures, which can therefore provide dedicated resources to execute remotely and privately the CoLoMoTo Jupyter web interface.

### 6.2. Extending the CoLoMoTo interactive notebook

The CoLoMoTo Docker image can be easily extended to include additional tools. The Docker architecture allows inheriting from an existing container, adding a new layer with additional executables. Contributions are welcome through GitHub^30^. Each software tool must be usable from the Jupyter interface and should be able to connect with at least one other tool already included. Furthermore, a demonstration notebook should be provided to illustrate the tool usage and how it can be combined with other tools.

Currently, all the embedded tools require an already defined model. Nevertheless, once loaded, a model can be subsequently modified from the Python interface (see tool feature matrix in Figure 1). We are currently considering the development of a programmatic interface for model definition *ab initio*. One of the main challenge is to provide a decent visualization of the programmatically-created model. A potential direction is to include a visual edition module in the Jupyter interface, which represents a substantial development effort.

The support for standard exchange formats is key to enable reproducibility of analyses with different tools. In that sense, bioLQM plays an important role for the CoLoMoTo Interactive Notebook as it provides bridges between SBML-qual standard specifications and numerous software tools (Figure 2). The Tellurium Notebook system by Sauro et al. (in review)^31^ offers support for SED-ML to help reproduce quantitative simulation of biological networks. Future work should consider bringing this feature for qualitative models as well, in order to better meet FAIR (Findability, Accessibility, Interoperability, and Reusability) recommendations (Wittig et al., 2017).

## Author contributions

AN, CH, DT, and LP designed the main principles of the CoLoMoTo Interactive notebook and its distribution. AN, CH, NL, and LP implemented the necessary Python modules, their integration in the Jupyter interface, and the Docker image. AN and LP edited the notebook tutorials, while CH edited the SnakeMake workflow example. All authors contributed to the writing of the article under the supervision of DT and LP.

All authors reviewed the content of this article and agreed to endorse it.

### Conflict of interest statement

The authors declare that the research was conducted in the absence of any commercial or financial relationships that could be construed as a potential conflict of interest.

## Footnotes

^1^International conference on Very Large Data Bases.

^2^ACM's Special Interest Group on Management Of Data.

^3^http://co.mbine.org

^4^http://colomoto.org

^5^The notebook can be previewed and downloaded at https://nbviewer.jupyter.org/github/colomoto/colomoto-docker/blob/2018-03-31/usecases/Usecase%20-%20Mutations%20enabling%20tumour%20invasion.ipynb

^6^Preprint on bioRxiv https://doi.org/10.1101/287011

^7^See https://docker.com for installation instructions.

^8^https://conda.io

^9^CoLoMoTo-related conda packages are available in the *colomoto* conda channel. See https://anaconda.org/colomoto

^10^https://pandas.pydata.org

^11^http://networkx.github.io

^12^http://ginsim.org/models_repository

^13^The notebook can be previewed and downloaded at https://nbviewer.jupyter.org/github/colomoto/colomoto-docker/blob/2018-03-31/tutorials/CellCollective/CellCollective%20-%20Knowledge%20Base.ipynb

^14^The notebook can be previewed and downloaded at https://nbviewer.jupyter.org/github/colomoto/colomoto-docker/blob/2018-03-31/tutorials/GINsim/GINsim%20-%20visualization.ipynb

^15^The notebook can be previewed and downloaded at https://nbviewer.jupyter.org/github/colomoto/colomoto-docker/blob/2018-03-31/tutorials/bioLQM/bioLQM_tutorial.ipynb

^16^The notebook can be previewed and downloaded at https://nbviewer.jupyter.org/github/colomoto/colomoto-docker/blob/2018-03-31/tutorials/Pint/quick-tutorial.ipynb

^17^The notebook can be previewed and downloaded at https://nbviewer.jupyter.org/github/colomoto/colomoto-docker/blob/2018-03-31/tutorials/NuSMV/NuSMV%20with%20GINsim.ipynb

^18^The notebook can be previewed and downloaded at https://nbviewer.jupyter.org/github/colomoto/colomoto-docker/blob/2018-03-31/tutorials/MaBoSS/MaBoSS%20-%20Quick%20tutorial.ipynb

^19^The notebook can be previewed and downloaded at https://nbviewer.jupyter.org/github/colomoto/colomoto-docker/blob/2018-03-31/tutorials/GINsim/GINsim%20-%20visualization.ipynb

^20^The notebook can be previewed and downloaded at https://nbviewer.jupyter.org/github/colomoto/colomoto-docker/blob/2018-03-31/tutorials/MaBoSS/Predict%20mutations%20with%20Pint,%20refine%20with%20MaBoSS.ipynb

^21^http://jupyter.org

^22^The notebook can be previewed and downloaded at https://nbviewer.jupyter.org/github/colomoto/colomoto-docker/blob/2018-03-31/tutorials/bioLQM/Fixpoints%20(interactive).ipynb

^23^The notebook can be previewed and downloaded at https://nbviewer.jupyter.org/github/colomoto/colomoto-docker/blob/2018-03-31/tutorials/Reproducibility%20-%20fixpoints.ipynb

^24^The notebook can be previewed and downloaded at https://nbviewer.jupyter.org/github/colomoto/colomoto-docker/blob/2018-03-31/tutorials/Reproducibility%20-%20model%20checking.ipynb

^25^You may have to use pip3 instead of pip depending on your configuration.

^26^If using Docker Toolbox, the command should be executed within the Docker Terminal.

^27^https://gist.github.com

^28^https://www.myexperiment.org

^29^https://zenodo.org

^30^Guidelines available at https://github.com/colomoto/colomoto-docker/blob/master/CONTRIBUTING.md

^31^Preprint on bioRxiv: https://doi.org/10.1101/239004

## Supplementary material

Data Sheet 1 The supplemental file “SnakeMake” contains an example of SnakeMake workflow that uses the CoLoMoTo Docker image to execute complementary analyses.

Data Sheet 2 The supplemental data “Notebooks” contains several short Jupyter notebooks which demonstrate different usage of the CoLoMoTo interactive notebook, listed in Table 2. The .ipynb files can be imported and executed within the Jupyter interface of the CoLoMoTo notebook, using the Docker image colomoto/colomoto-docker:2018-03-31. For each of these notebooks, a static HTML file previews the Jupyter rendering of the notebook, without any requirement. These notebooks can also be previewed and downloaded at https://nbviewer.jupyter.org/github/colomoto/colomoto-docker/tree/2018-03-31/tutorials.

## References

[B1] Abou-Jaoudé W. Monteiro P. T. Naldi A. Grandclaudon M. Soumelis V. Chaouiya C.. (2015). Model checking to assess t-helper cell plasticity. Front. Bioeng. Biotechnol. 2:86. 10.3389/fbioe.2014.00086 25674559 PMC4309205

[B2] Abou-Jaoudé W. Traynard P. Monteiro P. T. Saez-Rodriguez J. Helikar T. Thieffry D.. (2016). Logical modeling and dynamical analysis of cellular networks. Front. Genet. 7:94. 10.3389/fgene.2016.00094 27303434 PMC4885885

[B3] Albert I. Thakar J. Li S. Zhang R. Albert R. (2008). Boolean network simulations for life scientists. Source Code Biol. Med. 3:16. 10.1186/1751-0473-3-16 19014577 PMC2603008

[B4] Baker M. (2016). 1,500 scientists lift the lid on reproducibility. Nat. News 533:452. 10.1038/533452a 27225100

[B5] Bartocci E Lió P. (2016). Computational modeling, formal analysis, and tools for systems biology. PLOS Comput. Biol. 12:e1004591. 10.1371/journal.pcbi.1004591 26795950 PMC4721667

[B6] Batt G. Ropers D. de Jong H. Geiselmann J. Mateescu R. Page M.. (2005). Validation of qualitative models of genetic regulatory networks by model checking: analysis of the nutritional stress response in Escherichia coli. Bioinformatics 21(Suppl. 1), i19–i28. 10.1093/bioinformatics/bti1048 15961457

[B7] Begley C. G. Ellis L. M. (2012). Drug development: raise standards for preclinical cancer research. Nature 483, 531–533. 10.1038/483531a 22460880

[B8] Begley C. G. Ioannidis J. P. (2015). Reproducibility in science improving the standard for basic and preclinical research. Circ. Res. 116, 116–126. 10.1161/CIRCRESAHA.114.303819 25552691

[B9] Chaouiya C. Bérenguier D. Keating S. M. Naldi A. van Iersel M. P. Rodriguez N.. (2013). SBML qualitative models: a model representation format and infrastructure to foster interactions between qualitative modelling formalisms and tools. BMC Syst. Biol. 7:135. 10.1186/1752-0509-7-135 24321545 PMC3892043

[B10] Chaouiya C. Keating S. M. Berenguier D. Naldi A. Thieffry D. van Iersel M. P.. (2015). The Systems Biology Markup Language (SBML) level 3 package: qualitative models, version 1, release 1. J. Integr. Bioinform. 12:270. 10.1515/jib-2015-270 26528568

[B11] Cimatti A. Clarke E. Giunchiglia E. Giunchiglia F. Pistore M. Roveri M. (2002). NuSMV Version 2: an OpenSource Tool for Symbolic Model Checking, in Proceedings of International Conference on Computer-Aided Verification (CAV 2002), Vol. 2404 of LNCS (Copenhagen: Springer). 10.1007/3-540-45657-0_29

[B12] Cohen-Boulakia S. Belhajjame K. Collin O. Chopard J. Froidevaux C. Gaignard A. (2017). Scientific workflows for computational reproducibility in the life sciences: status, challenges and opportunities. Fut. Gen. Comput. Syst. 75, 284–298. 10.1016/j.future.2017.01.012

[B13] Drummond C. (2009). Replicability is not reproducibility: nor is it good science, in Proceedings of the Evaluation Methods for Machine Learning Workshop at the 26th ICML (Montreal, QC).

[B14] Errington T. M. Iorns E. Gunn W. Tan F. E. Lomax J. Nosek B. A. (2014). An open investigation of the reproducibility of cancer biology research. Elife 3:e04333. 10.7554/eLife.04333 25490932 PMC4270077

[B15] Fauré A. Naldi A. Chaouiya C. Thieffry D. (2006). Dynamical analysis of a generic boolean model for the control of the mammalian cell cycle. Bioinformatics 22, 124–31. 10.1093/bioinformatics/btl210 16873462

[B16] Freire J. Bonnet P. Shasha D. (2012). Computational reproducibility: state-of-the-art, challenges, and database research opportunities, in Proceedings of the 2012 ACM SIGMOD International Conference on Management of Data (Scottsdale, AZ), 593–596. 10.4230/DagRep.6.1.108

[B17] Freire J. Fuhr N. Rauber A. (2016). Reproducibility of data-oriented experiments in e-science, in Dagstuhl Seminar 16041 (Dagstuhl), 108–159.

[B18] Gan X. Albert R. (2018). General method to find the attractors of discrete dynamic models of biological systems. Phys. Rev. E 97:042308. 10.1103/PhysRevE.97.042308 29758614

[B19] Garg A. Di Cara A. Xenarios I. Mendoza L. De Micheli G. (2008). Synchronous versus asynchronous modeling of gene regulatory networks. Bioinformatics 24, 1917–1925. 10.1093/bioinformatics/btn336 18614585 PMC2519162

[B20] Goble C. A. Bhagat J. Aleksejevs S. Cruickshank D. Michaelides D. Newman D.. (2010). myExperiment: a repository and social network for the sharing of bioinformatics workflows. Nucleic Acids Res. 38 (Suppl. 2), W677–W682. 10.1093/nar/gkq429 20501605 PMC2896080

[B21] Goodman S. N. Fanelli D. Ioannidis J. P. (2016). What does research reproducibility mean? Sci. Transl. Med. 8:341ps12. 10.1126/scitranslmed.aaf5027 27252173

[B22] Helikar T. Kowal B. McClenathan S. Bruckner M. Rowley T. Madrahimov A.. (2012). The Cell Collective: toward an open and collaborative approach to systems biology. BMC Syst. Biol. 6:96. 10.1186/1752-0509-6-96 22871178 PMC3443426

[B23] Hucka M. Finney A. Sauro H. M. Bolouri H. Doyle J. C. Kitano H.. (2003). The Systems Biology Markup Language (SBML): a medium for representation and exchange of biochemical network models. Bioinformatics 19, 524–531. 10.1093/bioinformatics/btg015 12611808

[B24] Kauffman S. A. (1969). Metabolic stability and epigenesis in randomly constructed genetic nets. J. Theor. Biol. 22, 437–467. 10.1016/0022-5193(69)9001 5803332

[B25] Klarner H. Streck A. Siebert H. (2017). PyBoolNet: a python package for the generation, analysis and visualization of Boolean networks. Bioinformatics 33, 770–772. 10.1093/bioinformatics/btw682 27797783

[B26] Köster J. Rahmann S. (2012). Snakemake - a scalable bioinformatics workflow engine. Bioinformatics 28, 2520–2522. 10.1093/bioinformatics/bts480 22908215

[B27] Le Novère N. Finney A. Hucka M. Bhalla U. S. Campagne F. Collado-Vides J.. (2005). Minimum information requested in the annotation of biochemical models (MIRIAM). Nat. Biotechnol. 23, 1509–1515. 10.1038/nbt1156 16333295

[B28] Levy N. Naldi A. Hernandez C. Stoll G. Thieffry D. Zinovyev A. (2018). Prediction of mutations to control pathways enabling tumour cell invasion with the CoLoMoTo interactive notebook (tutorial). Front. Physiol. 9:787 10.3389/fphys.2018.00787 PMC6043725 30034343

[B29] Lewis J. Breeze C. E. Charlesworth J. MacLaren O. J. Cooper J. (2016). Where next for the reproducibility agenda in computational biology? BMC Syst. Biol. 10:52. 10.1186/s12918-016-0288-x 27422148 PMC4946111

[B30] Müssel C. Hopfensitz M. Kestler H. (2010). BoolNet–an R package for generation, reconstruction and analysis of Boolean networks. Bioinformatics 26, 1378–1380. 10.1093/bioinformatics/btq124 20378558

[B31] Naldi A. Hernandez C. Abou-Jaoudé W. Monteiro P. T. Chaouiya C. Thieffry D. (2018). Logical modelling and analysis of cellular regulatory networks with GINsim 3.0. Front. Physiol. 9:646 10.3389/fphys.2018.00646 PMC6018412 29971008

[B32] Naldi A. Monteiro P. T. Müssel C. Consortium for Logical Models Tools Kestler H. A. Thieffry D. Xenarios I.. (2015). Cooperative development of logical modelling standards and tools with CoLoMoTo. Bioinformatics 31, 1154–1159. 10.1093/bioinformatics/btv013 25619997

[B33] Naldi A. Remy E. Thieffry D. Chaouiya C. (2011). Dynamically consistent reduction of logical regulatory graphs. Theor. Comput. Sci. 412, 2207–2218. 10.1016/j.tcs.2010.10.021

[B34] Ostrowski M. Paulevé L. Schaub T. Siegel A. Guziolowski C. (2016). Boolean network identification from perturbation time series data combining dynamics abstraction and logic programming. Biosystems 149, 139–153. 10.1016/j.biosystems.2016.07.009 27484338

[B35] Paulevé L. (2017). Pint: a static analyzer for transient dynamics of qualitative networks with IPython interface, in CMSB 2017 - 15th Conference on Computational Methods for Systems Biology Volume 10545 of Lecture Notes in Computer Science (Darmstadt: Springer), 370–316. 10.1007/978-3-319-67471-1_20

[B36] Paulevé L. (in press). Reduction of qualitative models of biological networks for transient dynamics analysis. IEEE/ACM Trans. Comput. Biol. Bioinform. 10.1109/TCBB.2017.2749225. 28885158

[B37] Peng R. D. (2009). Reproducible research and biostatistics. Biostatistics 10, 405–408. 10.1093/biostatistics/kxp014 19535325

[B38] Ragan-Kelley M. Perez F. Granger B. Kluyver T. Ivanov P. Frederic J. (2014). The Jupyter/IPython architecture: a unified view of computational research, from interactive exploration to communication and publication, in AGU Fall Meeting Abstracts (San Francisco, CA).

[B39] Richter S. H. Garner J. P. Auer C. Kunert J. Würbel H. (2010). Systematic variation improves reproducibility of animal experiments. Nat. Methods 7, 167–168. 10.1038/nmeth0310-167 20195246

[B40] Santori G. (2016). Journals should drive data reproducibility. Nature 535, 355–355. 10.1038/535355b 27443731

[B41] Siebert H. (2009). Deriving behavior of boolean bioregulatory networks from subnetwork dynamics. Math. Comput. Sci. 2, 421–442. 10.1007/s11786-008-0064-4

[B42] Smith M. A. Houghton P. (2013). A proposal regarding reporting of *in vitro* testing results. Clin. Cancer Res. 19, 2828–2833. 10.1158/1078-0432.CCR-13-0043 23580781 PMC3741962

[B43] Stodden V. Guo P. Ma Z. (2013). Toward reproducible computational research: an empirical analysis of data and code policy adoption by journals. PLoS ONE 8:e67111. 10.1371/journal.pone.0067111 23805293 PMC3689732

[B44] Stodden V. Leisch F. Peng R. D. (2014). Implementing Reproducible Research. CRC Press.

[B45] Stoll G. Caron B. Viara E. Dugourd A. Zinovyev A. Naldi A.. (2017). MaBoSS 2.0: an environment for stochastic Boolean modeling. Bioinformatics 33, 2226–2228. 10.1093/bioinformatics/btx123 28881959

[B46] Stoll G. Viara E. Barillot E. Calzone L. (2012). Continuous time boolean modeling for biological signaling: application of gillespie algorithm. BMC Systems Biology 6:116. 10.1186/1752-0509-6-116 22932419 PMC3517402

[B47] Talcott C. Dill D. L. (2006). Multiple representations of biological processes, in Transactions on Computational Systems Biology VI, eds Priami C. Plotkin G. (Berlin; Heidelberg: Springer Science Business Media), 221–245. 10.1007/11880646_10

[B48] Terfve C. Cokelaer T. Henriques D. MacNamara A. Goncalves E. Morris M. K.. (2012). CellNOptR: a flexible toolkit to train protein signaling networks to data using multiple logic formalisms. BMC Syst. Biol. 6:133. 10.1186/1752-0509-6-133 23079107 PMC3605281

[B49] Thomas R. (1973). Boolean formalization of genetic control circuits. J. Theor. Biol. 42, 563–585. 10.1016/0022-5193(73)90247-6 4588055

[B50] Todd R. G. Helikar T. (2012). Ergodic sets as cell phenotype of budding yeast cell cycle. PLoS ONE 7:e45780. 10.1371/journal.pone.0045780 23049686 PMC3462196

[B51] Traynard P. Fauré A. Fages F. Thieffry D. (2016). Logical model specification aided by model-checking techniques: application to the mammalian cell cycle regulation. Bioinformatics 32, i772–i780. 10.1093/bioinformatics/btw457 27587700

[B52] Veliz-Cuba A. Aguilar B. Hinkelmann F. Laubenbacher R. (2014). Steady state analysis of boolean molecular network models via model reduction and computational algebra. BMC Bioinformatics 15:221. 10.1186/1471-2105-15-221 24965213 PMC4230806

[B53] Waltemath D. Adams R. Beard D. A. Bergmann F. T. Bhalla U. S. Britten R.. (2011a). Minimum Information About a Simulation Experiment (MIASE). PLoS Comput. Biol. 7:e1001122. 10.1371/journal.pcbi.1001122 21552546 PMC3084216

[B54] Waltemath D. Adams R. Bergmann F. T. Hucka M. Kolpakov F. Miller A. K.. (2011b). Reproducible computational biology experiments with SED-ML – the Simulation Experiment Description Markup Language. BMC Syst. Biol. 5:198. 10.1186/1752-0509-5-198 22172142 PMC3292844

[B55] Wittig U. Rey M. Weidemann A. Müller W. (2017). Data management and data enrichment for systems biology projects. J. Biotechnol. 261, 229–237. 10.1016/j.jbiotec.2017.06.007 28606610

[B56] Yaffe M. B. (2015). Reproducibility in science. Sci. Signal. 8:eg5. 10.1126/scisignal.aaa5764 25852185

