# Supplementary material for: The CoLoMoTo Interactive Notebook: Accessible and Reproducible Computational Analyses for Qualitative Biological Networks
Source: Front Physiol. 2018 Jun 19;9:680. doi: 10.3389/fphys.2018.00680 (PMC6018415; doi:10.3389/fphys.2018.00680)
Supplement: Data Sheet 2 — The supplemental data “Notebooks” contains several short Jupyter notebooks which demonstrate different usage of the CoLoMoTo interactive notebook, listed in Table 2. The .ipynb files can be imported and executed within the Jupyter interface of the CoLoMoTo notebook, using the Docker image colomoto/colomoto-docker:2018-03-31. For each of these notebooks, a static HTML file previews the Jupyter rendering of the notebook, without any requirement. These notebooks can also be previewed and downloaded at https://nbviewer.jupyter.org/github/colomoto/colomoto-docker/tree/2018-03-31/tutorials. [file Data_Sheet_2.ZIP › Notebooks/demo-reproducibility-fixpoints.html]

Reproducibility - fixpoints


In this notebook, we show how the computational of the fixpoints of qualitative regulatory networks can be done with different methods, which should give equivalent results, using *GINsim* and *Pint*.

### Model loading¶

We load a simple model available on http://ginsim.org/node/41

In [1]:

```
import pandas as pd # for displaying list of fixpoints
import ginsim
```

This notebook has been executed using the docker image `colomoto/colomoto-docker:2018-03-31`

In [2]:

```
th17 = ginsim.load("http://ginsim.org/sites/default/files/Th_17.zginml")
```

Downloading 'http://ginsim.org/sites/default/files/Th\_17.zginml'

### Computation of fixpoints with bioLQM¶

In [3]:

```
import biolqm
```

In [4]:

```
th17_lqm = ginsim.to_biolqm(th17)
```

In [5]:

```
fps_lqm = biolqm.fixpoints(th17_lqm)
pd.DataFrame(fps_lqm)
```

Out[5]:

|  | GATA3 | IFNb | IFNbR | IFNg | IFNgR | IL12 | IL12R | IL18 | IL18R | IL4 | IL4R | IRAK | SOCS1 | STAT1 | STAT4 | STAT6 | Tbet |
| --- | --- | --- | --- | --- | --- | --- | --- | --- | --- | --- | --- | --- | --- | --- | --- | --- | --- |
| 0 | 0 | 0 | 0 | 0 | 0 | 0 | 0 | 0 | 0 | 0 | 0 | 0 | 0 | 0 | 0 | 0 | 0 |
| 1 | 1 | 0 | 0 | 0 | 0 | 0 | 0 | 0 | 0 | 1 | 1 | 0 | 0 | 0 | 0 | 1 | 0 |
| 2 | 0 | 0 | 0 | 1 | 1 | 0 | 0 | 0 | 0 | 0 | 0 | 0 | 1 | 1 | 0 | 0 | 1 |
| 3 | 0 | 0 | 0 | 2 | 1 | 0 | 0 | 0 | 0 | 0 | 0 | 0 | 1 | 1 | 0 | 0 | 2 |

### Computation of fixpoints with Pint¶

In [6]:

```
import pypint
```

You are using Pint version 2018-03-22 and pypint 1.4.1

In [7]:

```
th17_an = biolqm.to_pint(th17_lqm)
```

In [8]:

```
fps_an = pypint.fixpoints(th17_an)
pd.DataFrame(fps_an)
```

Out[8]:

|  | GATA3 | IFNb | IFNbR | IFNg | IFNgR | IL12 | IL12R | IL18 | IL18R | IL4 | IL4R | IRAK | SOCS1 | STAT1 | STAT4 | STAT6 | Tbet |
| --- | --- | --- | --- | --- | --- | --- | --- | --- | --- | --- | --- | --- | --- | --- | --- | --- | --- |
| 0 | 0 | 0 | 0 | 0 | 0 | 0 | 0 | 0 | 0 | 0 | 0 | 0 | 0 | 0 | 0 | 0 | 0 |
| 1 | 1 | 0 | 0 | 0 | 0 | 0 | 0 | 0 | 0 | 1 | 1 | 0 | 0 | 0 | 0 | 1 | 0 |
| 2 | 0 | 0 | 0 | 1 | 1 | 0 | 0 | 0 | 0 | 0 | 0 | 0 | 1 | 1 | 0 | 0 | 1 |
| 3 | 0 | 0 | 0 | 2 | 1 | 0 | 0 | 0 | 0 | 0 | 0 | 0 | 1 | 1 | 0 | 0 | 2 |

### Display fixpoint using GINsim¶

In [9]:

```
ginsim.show(th17, fps_lqm[1]) # or fps_an[1]
```

Out[9]:
