# Supplementary material for: The CoLoMoTo Interactive Notebook: Accessible and Reproducible Computational Analyses for Qualitative Biological Networks
Source: Front Physiol. 2018 Jun 19;9:680. doi: 10.3389/fphys.2018.00680 (PMC6018415; doi:10.3389/fphys.2018.00680)
Supplement: Data Sheet 2 — The supplemental data “Notebooks” contains several short Jupyter notebooks which demonstrate different usage of the CoLoMoTo interactive notebook, listed in Table 2. The .ipynb files can be imported and executed within the Jupyter interface of the CoLoMoTo notebook, using the Docker image colomoto/colomoto-docker:2018-03-31. For each of these notebooks, a static HTML file previews the Jupyter rendering of the notebook, without any requirement. These notebooks can also be previewed and downloaded at https://nbviewer.jupyter.org/github/colomoto/colomoto-docker/tree/2018-03-31/tutorials. [file Data_Sheet_2.ZIP › Notebooks/demo-ginsim.html]

GINsim - visualization


## Regulatory graph visualization with GINsim¶

GINsim models in GINML format contains layout information for displaying the regulatory graph of a Boolean or multi-valued network. The regulatory graph shows the activation and inhibition relations between the nodes of the network.

In this notebook, we show how to use the `ginsim` Python module to visualize a network model, with optionnaly a state.

In [1]:

```
import ginsim
```

This notebook has been executed using the docker image `colomoto/colomoto-docker:2018-03-31`

First, we load a model from the GINsim repository:

In [2]:

```
lrg = ginsim.load("http://ginsim.org/sites/default/files/SuppMat_Model_Master_Model.zginml")
```

Downloading 'http://ginsim.org/sites/default/files/SuppMat\_Model\_Master\_Model.zginml'

The regulatory graph can be visualized using the `ginsim.show` function:

In [3]:

```
ginsim.show(lrg)
```

Out[3]:

The `show` function also allows an additional parameters which specifies for each node a state value (0 or 1 in case of a Boolean node).
This state can also come from the result of a model analysis, for instance, a fixpoint analysis.

Let us use biolqm to compute the fixpoints of the model with bioLQM:

In [4]:

```
import biolqm
```

First, we convert the model to bioLQM:

In [5]:

```
lqm = ginsim.to_biolqm(lrg)
```

Then, we use `biolqm.fixpoints` to compute the list of all the fixpoints of the network, and store it in the `fps` variable:

In [6]:

```
fps = biolqm.fixpoints(lqm)
print(len(fps), "fixpoints")
```

```
9 fixpoints
```

The third fixpoint (`fps[2]`) can then be displayed as follows:

In [7]:

```
ginsim.show(lrg, fps[2])
```

Out[7]:
