# Supplementary material for: The CoLoMoTo Interactive Notebook: Accessible and Reproducible Computational Analyses for Qualitative Biological Networks
Source: Front Physiol. 2018 Jun 19;9:680. doi: 10.3389/fphys.2018.00680 (PMC6018415; doi:10.3389/fphys.2018.00680)
Supplement: Data Sheet 2 — The supplemental data “Notebooks” contains several short Jupyter notebooks which demonstrate different usage of the CoLoMoTo interactive notebook, listed in Table 2. The .ipynb files can be imported and executed within the Jupyter interface of the CoLoMoTo notebook, using the Docker image colomoto/colomoto-docker:2018-03-31. For each of these notebooks, a static HTML file previews the Jupyter rendering of the notebook, without any requirement. These notebooks can also be previewed and downloaded at https://nbviewer.jupyter.org/github/colomoto/colomoto-docker/tree/2018-03-31/tutorials. [file Data_Sheet_2.ZIP › Notebooks/demo-nusmv.html]

NuSMV with GINsim


This notebooks shows a basic example of using NuSMV to analyse dynamical properties on a GINsim model.

## Model¶

We use GINsim to load a simple model of phage lambda differentiation.

In [1]:

```
import ginsim
```

This notebook has been executed using the docker image `colomoto/colomoto-docker:2018-03-31`

In [2]:

```
lrg = ginsim.load("http://ginsim.org/sites/default/files/phageLambda4.zginml")
```

Downloading 'http://ginsim.org/sites/default/files/phageLambda4.zginml'

## Dynamical properties¶

The phage lambda model exhibits the differentiation process between lytic and lysogenic states.

In this notebook, we will CTL to express properties on trajectories related to the reachability of lytic and lysogenic attractors.

The Python module `colomoto.temporal_logics` provides a programmatic way to specify CTL and LTL properties

In [3]:

```
from colomoto.temporal_logics import *
```

Properties on states are specified using the `S` operator.
The following property characterize the initial state when all the nodes are inactive:

In [4]:

```
initial_state = S(CI=0,CII=0,Cro=0,N=0)
```

The lysogenic state is characterized by the node `CI` being permanently strongly expressed, which is modeled by `CI=2`. The following CTL property express that from the given state, all the reachable states (`AG`) satisfy `CI=2`:

In [5]:

```
lysogenic = AG(S(CI=2))
```

The lytic state is characterized by the node `CI` being inactive (value 0), and `Cro` oscillating between activity levels 2 and 3. This is stated by the following CTL property:

In [6]:

```
lytic = AG(S(CI=0) & (S(Cro=2) | S(Cro=3)))
```

The following CTL properties is true if all the reachable attractors are either lysogenic or lytic states:

In [7]:

```
attractors = AG(EF(lysogenic | lytic))
```

## Setting up NuSMV¶

NuSMV is a symbolic model checker which checks if the provided model verifies a given set of CTL/LTL properties.

GINsim provides a translation of Boolean and multivalued networks into NuSMV models:

In [8]:

```
smv = ginsim.to_nusmv(lrg)
```

Here, `smv` is a Python object representing the NuSMV model.
CTL/LTL specification can be added using the method `add_ctl` and `add_ltl`, respectively.
For convenience, a label can be given to a property:

In [9]:

```
smv.add_ctl(attractors, name="global_attractors")
```

Properties can also be added by bulk, providing a Python dictionnary specifying the properties.
In the following cell, we specify two properties: one verifying that, from the initial state, there exists a trajectory leading to a stable lysogenic state, and another one verifying that, from the initial state, there exists a trajectory leading to a stable lytic state.

In [10]:

```
specs = {
    "reach_lyso": If(initial_state, EF(lysogenic)),
    "reach_lytic": If(initial_state, EF(lytic))
}
```

In [11]:

```
smv.add_ctls(specs)
```

## Invoking NuSMV¶

The method `verify` will execute NuSMV and returns the verification result for each property:

In [12]:

```
smv.verify()
```

Out[12]:

```
{'global_attractors': True, 'reach_lyso': True, 'reach_lytic': True}
```

One can also use the method `alltrue` to check if all the given properties are true:

In [13]:

```
smv.alltrue()
```

Out[13]:

```
True
```
