# Supplementary material for: The CoLoMoTo Interactive Notebook: Accessible and Reproducible Computational Analyses for Qualitative Biological Networks
Source: Front Physiol. 2018 Jun 19;9:680. doi: 10.3389/fphys.2018.00680 (PMC6018415; doi:10.3389/fphys.2018.00680)
Supplement: Data Sheet 2 — The supplemental data “Notebooks” contains several short Jupyter notebooks which demonstrate different usage of the CoLoMoTo interactive notebook, listed in Table 2. The .ipynb files can be imported and executed within the Jupyter interface of the CoLoMoTo notebook, using the Docker image colomoto/colomoto-docker:2018-03-31. For each of these notebooks, a static HTML file previews the Jupyter rendering of the notebook, without any requirement. These notebooks can also be previewed and downloaded at https://nbviewer.jupyter.org/github/colomoto/colomoto-docker/tree/2018-03-31/tutorials. [file Data_Sheet_2.ZIP › Notebooks/demo-biolqm.html]

bioLQM\_tutorial


# BioLQM tutorial¶

This notebook illustrates the main features of the python API of the `biolqm` toolkit. We will show how to load a model, perform simple dynamical analysis (simulation and identification of attractors) and apply perturbations.

The code from this tutorial can be executed on the standard python interpreter, the IPython shell or the Jupyter notebook web interface.

In [1]:

```
import pandas as pd # for the visualization of lists of states
import biolqm
```

This notebook has been executed using the docker image `colomoto/colomoto-docker:2018-03-31`

## Load a model¶

The `biolqm.load` function allows to load a model froma file given as parameter. It can be a local file or a web URL to downloadit. bioLQM will use the file extension to guess the format.
See https://github.com/colomoto/bioLQM#how-to-use-it for a list of supported formats, notably GINsim (`.zginml` or `.ginml` files) and SBML qual (`.sbml` files).

When using the web interface, calling the load function without specifying the parameter enables to pick and upload a local file.

Here we load a published model of the mammalian cell cycle.

In [2]:

```
lqm = biolqm.load("http://ginsim.org/sites/default/files/boolean_cell_cycle.zginml")
```

Downloading 'http://ginsim.org/sites/default/files/boolean\_cell\_cycle.zginml'

# Deterministic simulations¶

Starting with an initial state (by default where all components are inactive), we can compute the evolution of all components over time by evaluating the logical functions of the model. The `biolqm.trace` function returns an iterator from which we can obtain a list of successive states.

These simulations are limited to *deterministic* updating modes, where each state has a single successor.
The simulation stops when reaching a stable state or a maximal number of steps (1000 by default).

In [3]:

```
trace = biolqm.trace(lqm)
pd.DataFrame( [s for s in trace] )
```

Out[3]:

|  | Cdc20 | CycA | CycB | CycD | CycE | E2F | Rb | UbcH10 | cdh1 | p27 |
| --- | --- | --- | --- | --- | --- | --- | --- | --- | --- | --- |
| 0 | 0 | 0 | 0 | 0 | 0 | 0 | 0 | 0 | 0 | 0 |
| 1 | 0 | 0 | 1 | 0 | 0 | 1 | 1 | 1 | 1 | 1 |
| 2 | 1 | 0 | 0 | 0 | 0 | 0 | 0 | 1 | 0 | 0 |
| 3 | 0 | 0 | 0 | 0 | 0 | 1 | 1 | 1 | 1 | 1 |
| 4 | 0 | 0 | 0 | 0 | 0 | 0 | 1 | 0 | 1 | 1 |

The `biolqm.trace` function can take an additional argument to specify simulation parameters. These parameters are specified in a single string and identified by the following flags:

- `-u` for the updating mode (synchronous by default, can also be sequential)
- `-i` for the initial state (following the internal component ordering)
- `-m` for the maximal number of steps.

In [4]:

```
trace = biolqm.trace(lqm, "-u synchronous -i 0010000000 -m 50")
pd.DataFrame( [s for s in trace] )
```

Out[4]:

|  | Cdc20 | CycA | CycB | CycD | CycE | E2F | Rb | UbcH10 | cdh1 | p27 |
| --- | --- | --- | --- | --- | --- | --- | --- | --- | --- | --- |
| 0 | 0 | 0 | 0 | 0 | 0 | 1 | 0 | 0 | 0 | 0 |
| 1 | 0 | 1 | 1 | 0 | 1 | 1 | 1 | 1 | 1 | 1 |
| 2 | 1 | 0 | 0 | 0 | 0 | 0 | 0 | 1 | 0 | 0 |
| 3 | 0 | 0 | 0 | 0 | 0 | 1 | 1 | 1 | 1 | 1 |
| 4 | 0 | 0 | 0 | 0 | 0 | 0 | 1 | 0 | 1 | 1 |

# Random walk in non-deterministic simulations¶

Logical models can also be updated in a *non-deterministic* mode, where each state can have many alternative successors.
The `biolqm.random` function allows to perform a random walk in the complex dynamics obtained with non-deterministic updating modes.

Like `biolqm.trace`, the `biolqm.random` function takes an additional argument for simulation parameters:

- `-u` for the updating mode (asynchronous by default, can also be complete)
- `-i` for the initial state (following the internal component ordering)
- `-m` for the maximal number of steps.

In [5]:

```
random = biolqm.random(lqm, "-i 0010000000 -m 50")
pd.DataFrame( [s for s in random] )
```

Out[5]:

|  | Cdc20 | CycA | CycB | CycD | CycE | E2F | Rb | UbcH10 | cdh1 | p27 |
| --- | --- | --- | --- | --- | --- | --- | --- | --- | --- | --- |
| 0 | 0 | 0 | 0 | 0 | 0 | 1 | 0 | 0 | 0 | 0 |
| 1 | 0 | 1 | 0 | 0 | 0 | 1 | 0 | 0 | 0 | 0 |
| 2 | 0 | 1 | 0 | 0 | 0 | 1 | 0 | 1 | 0 | 0 |
| 3 | 0 | 1 | 0 | 0 | 0 | 0 | 0 | 1 | 0 | 0 |
| 4 | 0 | 1 | 1 | 0 | 0 | 0 | 0 | 1 | 0 | 0 |
| 5 | 1 | 1 | 1 | 0 | 0 | 0 | 0 | 1 | 0 | 0 |
| 6 | 1 | 1 | 1 | 0 | 0 | 0 | 0 | 1 | 1 | 0 |
| 7 | 1 | 1 | 0 | 0 | 0 | 0 | 0 | 1 | 1 | 0 |
| 8 | 0 | 1 | 0 | 0 | 0 | 0 | 0 | 1 | 1 | 0 |
| 9 | 0 | 0 | 0 | 0 | 0 | 0 | 0 | 1 | 1 | 0 |
| 10 | 0 | 0 | 0 | 0 | 0 | 0 | 1 | 1 | 1 | 0 |
| 11 | 0 | 0 | 0 | 0 | 0 | 0 | 1 | 0 | 1 | 0 |
| 12 | 0 | 0 | 0 | 0 | 0 | 0 | 1 | 0 | 1 | 1 |

As this type of simulation is a random walk, successive calls often yield different trajectories.

In [6]:

```
pd.DataFrame( [s for s in random] )
```

Out[6]:

|  | Cdc20 | CycA | CycB | CycD | CycE | E2F | Rb | UbcH10 | cdh1 | p27 |
| --- | --- | --- | --- | --- | --- | --- | --- | --- | --- | --- |
| 0 | 0 | 0 | 0 | 0 | 0 | 1 | 0 | 0 | 0 | 0 |
| 1 | 0 | 0 | 0 | 0 | 0 | 1 | 0 | 1 | 0 | 0 |
| 2 | 0 | 0 | 0 | 0 | 0 | 1 | 1 | 1 | 0 | 0 |
| 3 | 0 | 0 | 1 | 0 | 0 | 1 | 1 | 1 | 0 | 0 |
| 4 | 1 | 0 | 1 | 0 | 0 | 1 | 1 | 1 | 0 | 0 |
| 5 | 1 | 0 | 1 | 0 | 0 | 0 | 1 | 1 | 0 | 0 |
| 6 | 1 | 0 | 0 | 0 | 0 | 0 | 1 | 1 | 0 | 0 |
| 7 | 1 | 0 | 0 | 0 | 0 | 0 | 1 | 1 | 1 | 0 |
| 8 | 1 | 0 | 0 | 0 | 0 | 0 | 1 | 1 | 1 | 1 |
| 9 | 0 | 0 | 0 | 0 | 0 | 0 | 1 | 1 | 1 | 1 |
| 10 | 0 | 0 | 0 | 0 | 0 | 0 | 1 | 0 | 1 | 1 |

While bioLQM implements non-deterministic updating modes, their simulation requires a more complex engine and data structure which are beyond the scope of bioLQM: use other tools such as GINsim or (py)boolnet to perform non-deterministic simulations.

## Identification of stable states (fixed points)¶

The `biolqm.fixpoints` functions implements a constraint-solving method (based on decision diagrams) for the efficient identification of the stable states of a model without performing simulation.

In [7]:

```
fps = biolqm.fixpoints(lqm)
pd.DataFrame(fps)
```

Out[7]:

|  | Cdc20 | CycA | CycB | CycD | CycE | E2F | Rb | UbcH10 | cdh1 | p27 |
| --- | --- | --- | --- | --- | --- | --- | --- | --- | --- | --- |
| 0 | 0 | 0 | 0 | 0 | 0 | 0 | 1 | 0 | 1 | 1 |

## Identification of stable motifs (trapspaces)¶

A stable motif (also called symbolic steady state) is a partially assigned state such that all possible successors of all states which belong to the motif also belong to the motif. Like stable states, these stable motifs can be identified efficiently using constraint-solving methods.

Following this definition, all stable motifs contain at least an attractor. The stable states and the full state space are trivially identified stable motifs. Note that some stable motifs are embded inside others (in particular, they are all part of the full state space). The stable motifs which do not contain such smaller sub-motifs are called *terminal* and provide a good approximation of the attractors of the model (yet some attractors may be missed by this approximation).

The `biolqm.trapspace` function computes these stable motifs. An additional argument allows to show only the terminal ones.

In the output below, the non-assigned components in each motif are denoted by the joker value `255`.

In [8]:

```
traps = biolqm.trapspace(lqm)
pd.DataFrame(traps)
```

Out[8]:

|  | Cdc20 | CycA | CycB | CycD | CycE | E2F | Rb | UbcH10 | cdh1 | p27 |
| --- | --- | --- | --- | --- | --- | --- | --- | --- | --- | --- |
| 0 | 255 | 255 | 255 | 255 | 255 | 255 | 255 | 255 | 255 | 255 |
| 1 | 255 | 255 | 255 | 1 | 255 | 255 | 0 | 255 | 255 | 0 |
| 2 | 255 | 255 | 255 | 0 | 255 | 255 | 255 | 255 | 255 | 255 |
| 3 | 0 | 0 | 0 | 0 | 0 | 0 | 1 | 0 | 1 | 1 |

In [9]:

```
traps = biolqm.trapspace(lqm, "terminal")
pd.DataFrame(traps)
```

Out[9]:

|  | Cdc20 | CycA | CycB | CycD | CycE | E2F | Rb | UbcH10 | cdh1 | p27 |
| --- | --- | --- | --- | --- | --- | --- | --- | --- | --- | --- |
| 0 | 255 | 255 | 255 | 1 | 255 | 255 | 0 | 255 | 255 | 0 |
| 1 | 0 | 0 | 0 | 0 | 0 | 0 | 1 | 0 | 1 | 1 |

## Model perturbation¶

the `biolqm.perturbation` function enables the construction of a variant of the model, where the logical function of one (or several) component has been modified. A textual parameter describes the modification:

- `component%0` defines a knockout of a component
- `component%1` defines an ectopic expression
- `component%1-2` restricts the range of values for multi-valued components
- `component:regulator%0` allows to remove a regulator

In the following, we show the impact of the ectopic expression of the `CycD` component on the stable states and trapspaces on the model.

In [10]:

```
pert = biolqm.perturbation(lqm, "CycD%1")
```

In [11]:

```
fps = biolqm.fixpoints(pert)
pd.DataFrame(fps)
```

Out[11]:

|  |
| --- |

In [12]:

```
traps = biolqm.trapspace(pert, "terminal")
pd.DataFrame(traps)
```

Out[12]:

|  | Cdc20 | CycA | CycB | CycD | CycE | E2F | Rb | UbcH10 | cdh1 | p27 |
| --- | --- | --- | --- | --- | --- | --- | --- | --- | --- | --- |
| 0 | 255 | 255 | 255 | 1 | 255 | 255 | 0 | 255 | 255 | 0 |

Multiple perturbations are comma-separated. Here we show the effect of the ectopic expression of both `CycD` and `Rb`.

In [13]:

```
pert = biolqm.perturbation(lqm, "CycD%1,Rb%1")
```

In [14]:

```
fps = biolqm.fixpoints(pert)
pd.DataFrame(fps)
```

Out[14]:

|  | Cdc20 | CycA | CycB | CycD | CycE | E2F | Rb | UbcH10 | cdh1 | p27 |
| --- | --- | --- | --- | --- | --- | --- | --- | --- | --- | --- |
| 0 | 0 | 0 | 0 | 1 | 0 | 0 | 1 | 0 | 1 | 0 |

In [15]:

```
traps = biolqm.trapspace(pert, "terminal")
pd.DataFrame(traps)
```

Out[15]:

|  | Cdc20 | CycA | CycB | CycD | CycE | E2F | Rb | UbcH10 | cdh1 | p27 |
| --- | --- | --- | --- | --- | --- | --- | --- | --- | --- | --- |
| 0 | 0 | 0 | 0 | 1 | 0 | 0 | 1 | 0 | 1 | 0 |
