# Supplementary material for: The CoLoMoTo Interactive Notebook: Accessible and Reproducible Computational Analyses for Qualitative Biological Networks
Source: Front Physiol. 2018 Jun 19;9:680. doi: 10.3389/fphys.2018.00680 (PMC6018415; doi:10.3389/fphys.2018.00680)
Supplement: Data Sheet 2 — The supplemental data “Notebooks” contains several short Jupyter notebooks which demonstrate different usage of the CoLoMoTo interactive notebook, listed in Table 2. The .ipynb files can be imported and executed within the Jupyter interface of the CoLoMoTo notebook, using the Docker image colomoto/colomoto-docker:2018-03-31. For each of these notebooks, a static HTML file previews the Jupyter rendering of the notebook, without any requirement. These notebooks can also be previewed and downloaded at https://nbviewer.jupyter.org/github/colomoto/colomoto-docker/tree/2018-03-31/tutorials. [file Data_Sheet_2.ZIP › Notebooks/demo-pint+maboss.html]

Predict mutations with Pint, refine with MaBoSS


This notebook shows a possible way to combine formal prediction of mutation performed by Pint with the quantiative evaluation of attractors reachability with MaBoSS.

A detailed use case of such an analysis can be found in the notebook "Usecase - Mutations enabling tumour invasion".

## Model¶

We load a model of cell fate decision model from CellCollective using bioLQM:

In [1]:

```
import biolqm
```

This notebook has been executed using the docker image `colomoto/colomoto-docker:2018-03-31`

In [2]:

```
lqm = biolqm.load("https://cellcollective.org/#5884/tumour-cell-invasion-and-migration")
```

Downloading 'http://api.cellcollective.org/model/export/5884?type=SBML'

## Wild-type simulation with MaBoSS¶

We convert the model to MaBoSS, and configure the simulation

In [3]:

```
import maboss
```

In [4]:

```
wt_sim = biolqm.to_maboss(lqm)
```

In [5]:

```
wt_sim.network.set_output(('Metastasis', 'Migration', 'Invasion', 'Apoptosis', 'CellCycleArrest'))
```

In [6]:

```
wt_sim.network.set_istate("ECM", [0, 1]) # ECM is active
wt_sim.network.set_istate("DNAdamage", [0.5, 0.5]) # DNAdamage can start either active or inactive
```

In [7]:

```
wt_sim.update_parameters(max_time=50)
```

We perform the simulation with MaBoSS, this can take several seconds.

In [8]:

```
wt_res = wt_sim.run()
```

We plot the distribution of attractors at the end of the simulations:

In [9]:

```
wt_res.plot_piechart()
```

## Mutation prediction with Pint¶

Now, we use Pint to predict mutations which remove any possible activation of apoptosis.

In [10]:

```
import pypint
```

You are using Pint version 2018-03-22 and pypint 1.4.1

In [11]:

```
m = biolqm.to_pint(lqm)
```

In [12]:

```
m.initial_state["ECM"] = 1
m.initial_state["DNAdamage"] = {0,1}
```

In [13]:

```
mutants = m.oneshot_mutations_for_cut("Apoptosis=1", exclude={"ECM", "DNAdamage"})
mutants
```

This computation is an *under-approximation*: returned mutations are all valid, but they may be non-minimal, and some solutions may be missed.

Limiting solutions to mutations of at most 5 automata. Use `maxsize` argument to change.

Out[13]:

```
[{'ZEB2': 1},
 {'AKT1': 1},
 {'AKT2': 1},
 {'ERK': 1},
 {'ZEB1': 1, 'miR203': 1, 'p53': 0},
 {'SNAI2': 1, 'ZEB1': 1, 'miR203': 1},
 {'ZEB1': 1, 'p53': 0, 'p63': 0},
 {'NICD': 1, 'ZEB1': 1, 'p53': 0},
 {'SNAI2': 1, 'ZEB1': 1, 'p63': 0},
 {'NICD': 1, 'SNAI2': 1, 'ZEB1': 1},
 {'miR203': 1, 'p53': 0, 'p73': 0},
 {'SNAI2': 1, 'miR203': 1, 'p73': 0},
 {'p53': 0, 'p63': 0, 'p73': 0},
 {'NICD': 1, 'p53': 0, 'p73': 0},
 {'SNAI2': 1, 'p63': 0, 'p73': 0},
 {'NICD': 1, 'SNAI2': 1, 'p73': 0}]
```

Each returned solution gives a combination of mutations which are guaranteed to remove the capability to activate apoptosis, even transiently.

## Collecting experiments¶

Among the results returned by Pint, we want to try any potential double-mutant that may be sufficient to remove the reachability of a *stable* apoptosis.

We use Python standard library functions to compute all the couple of mutations in each predicted mutations, and then merge these couples in a single set of candidate double-mutants.

In [14]:

```
from itertools import combinations
from functools import reduce
```

In [15]:

```
mutant_combinations = [combinations(m.items(), 2) for m in mutants if len(m) >= 2]
candidates = reduce(set.union, mutant_combinations, set())
candidates
```

Out[15]:

```
{(('NICD', 1), ('SNAI2', 1)),
 (('ZEB1', 1), ('NICD', 1)),
 (('ZEB1', 1), ('SNAI2', 1)),
 (('ZEB1', 1), ('miR203', 1)),
 (('ZEB1', 1), ('p53', 0)),
 (('ZEB1', 1), ('p63', 0)),
 (('miR203', 1), ('SNAI2', 1)),
 (('miR203', 1), ('p53', 0)),
 (('miR203', 1), ('p73', 0)),
 (('p53', 0), ('NICD', 1)),
 (('p53', 0), ('p63', 0)),
 (('p63', 0), ('SNAI2', 1)),
 (('p73', 0), ('NICD', 1)),
 (('p73', 0), ('SNAI2', 1)),
 (('p73', 0), ('p53', 0)),
 (('p73', 0), ('p63', 0))}
```

## Double-mutant experiments with MaBoSS¶

For each candidate double-mutant, we copy the MaBoSS wild-type model, apply the corresponding mutation and run the simulations. As there are 16 candidates to evaluate, the overall execution can take several minutes.

In [16]:

```
import matplotlib.pyplot as plt # for customizing the plots
```

In [17]:

```
for mutant in sorted(candidates):
    mut_sim = wt_sim.copy()
    for (node, value) in mutant:
        mut_sim.mutate(node, "ON" if value else "OFF")
    mut_res = mut_sim.run()
    mut_res.plot_piechart(embed_labels=False, autopct=4)
    mutant_name = "/".join(["%s:%s"%m for m in mutant])
    plt.title("%s mutant" % mutant_name)
```
