# Supplementary material for: The CoLoMoTo Interactive Notebook: Accessible and Reproducible Computational Analyses for Qualitative Biological Networks
Source: Front Physiol. 2018 Jun 19;9:680. doi: 10.3389/fphys.2018.00680 (PMC6018415; doi:10.3389/fphys.2018.00680)
Supplement: Data Sheet 2 — The supplemental data “Notebooks” contains several short Jupyter notebooks which demonstrate different usage of the CoLoMoTo interactive notebook, listed in Table 2. The .ipynb files can be imported and executed within the Jupyter interface of the CoLoMoTo notebook, using the Docker image colomoto/colomoto-docker:2018-03-31. For each of these notebooks, a static HTML file previews the Jupyter rendering of the notebook, without any requirement. These notebooks can also be previewed and downloaded at https://nbviewer.jupyter.org/github/colomoto/colomoto-docker/tree/2018-03-31/tutorials. [file Data_Sheet_2.ZIP › Notebooks/demo-maboss.html]

MaBoSS - Quick tutorial


# Stochastic simulation of Boolean network with MaBoSS¶

This notebook show the basic features of the `maboss` Python module by reproducing part of the official MaBoSS tutorial at https://maboss.curie.fr/pub/TutorialMaBoSS\_2\_0.pdf

In [1]:

```
import maboss
import ginsim
```

This notebook has been executed using the docker image `colomoto/colomoto-docker:2018-03-31`

### Model¶

The input model is a simple Boolean network created with GINsim. We download it from MaBoSS website and display its regulatory graph using the `ginsim` Python module:

In [2]:

```
lrg = ginsim.load("https://maboss.curie.fr/pub/example.zginml")
```

Downloading 'https://maboss.curie.fr/pub/example.zginml'

In [3]:

```
ginsim.show(lrg)
```

Out[3]:

### Building the `Simulation` object¶

A GINsim model can be converted to MaBoSS using the `to_maboss` function:

In [4]:

```
masim = ginsim.to_maboss(lrg)
```

MaBoSS allows to specify some nodes as "internal": the value of internal nodes is hidden in the output of simulations.
By default, (if the network was built from scratch or if the `.cfg` file did not contain the information for a given node), all nodes have the attribute `is_internal` set to `False`. This can be changed within the script

In [5]:

```
masim.network['DNAdam'].is_internal = True
```

You can change other MaBoSS parameters with the function update\_parameters:

In [6]:

```
masim.update_parameters(time_tick=0.1, max_time=4)
```

Once all the parameters are set, you can run the simulation. The following line will create two temporary files that contains everything MaBoSS needs to know to run properly. The result of the simulation are stored in a `Result` object.

To run the simulation, use the command below. This command can be quickly written by going to MaBoSS -> Simulation -> run.

### Performing simulations¶

The `run` method of the simulation object will call MaBoSS to perfom the requested simulations. A result object is then returned.

In [7]:

```
res = masim.run()
```

### Result visualizations¶

The result object provides access to the simulation output, and provides simple plotting functions.

The `plot_trajectory` method displays the mean probability of each state (composed of non-internal nodes) along time:

In [8]:

```
res.plot_trajectory()
```

The `plot_piechart` method displays the proportion of states in which each simulation ended, providing an approximation of the probability to reach the different attractors.

In [9]:

```
res.plot_piechart(autopct=True)
```

### Simulating mutations¶

The `maboss` Python module allow to modify a network model to perform the mutation of a node, that is forcing its value to be always 1 ("ON") or always 0 ("OFF").

In the following cell, we copy the initial model and configure a gain of function mutant for the node `Mdm2nuc`:

In [10]:

```
mutsim = masim.copy()
mutsim.mutate("Mdm2nuc", "ON")
```

The resulting model can then be simulated:

In [11]:

```
mutres = mutsim.run()
```

In [12]:

```
mutres.plot_piechart()
```
