# Supplementary material for: The CoLoMoTo Interactive Notebook: Accessible and Reproducible Computational Analyses for Qualitative Biological Networks
Source: Front Physiol. 2018 Jun 19;9:680. doi: 10.3389/fphys.2018.00680 (PMC6018415; doi:10.3389/fphys.2018.00680)
Supplement: Data Sheet 2 — The supplemental data “Notebooks” contains several short Jupyter notebooks which demonstrate different usage of the CoLoMoTo interactive notebook, listed in Table 2. The .ipynb files can be imported and executed within the Jupyter interface of the CoLoMoTo notebook, using the Docker image colomoto/colomoto-docker:2018-03-31. For each of these notebooks, a static HTML file previews the Jupyter rendering of the notebook, without any requirement. These notebooks can also be previewed and downloaded at https://nbviewer.jupyter.org/github/colomoto/colomoto-docker/tree/2018-03-31/tutorials. [file Data_Sheet_2.ZIP › Notebooks/demo-reproducibility-modelchecking.html]

Reproducibility - model checking


In this notebook, we show how the verification of qualitative regulatory networks can be done with different methods, which should give equivalent results, using *GINsim* and *Pint*.

### Model loading¶

We load a simple model of Phage Lambda using GINsim, http://ginsim.org/node/47:

In [1]:

```
import ginsim
```

This notebook has been executed using the docker image `colomoto/colomoto-docker:2018-03-31`

In [2]:

```
lrg = ginsim.load("http://ginsim.org/sites/default/files/phageLambda4.zginml")
```

Downloading 'http://ginsim.org/sites/default/files/phageLambda4.zginml'

### Properties to be verified¶

We use the `colomoto` python module which offers a generic interface for declaring temporal properties using either LTL or CTL.

In [3]:

```
from colomoto.temporal_logics import *
```

In [4]:

```
lysogenic = AG(S(CI=2))        # CI is permanently active
lytic = AG(EF(S(CI=0,Cro=2)) & EF(S(CI=0,Cro=3)))  # Cro permanently oscillates between levels 2 and 3
attractors = AG(EF(lysogenic | lytic))   # all the attractors are either lysogenic or lytic
initial_state = S(CI=0,CII=0,Cro=0,N=0)
```

In [5]:

```
properties = {
    "s0_lysogenic": If(initial_state, EF(lysogenic)), # lysogenic state is reachable from initial state  
    "s0_lytic": If(initial_state, EF(lytic)),  # lytic state is reachable from initial state
    "attractors": attractors, # all attractors are either lyso or lytic
}
```

### Verification using GINsim export to NuSMV¶

In [6]:

```
smv_ginsim = ginsim.to_nusmv(lrg)
smv_ginsim.add_ctls(properties)
smv_ginsim.alltrue()
```

Out[6]:

```
True
```

### Verification using Pint export to NuSMV¶

In [7]:

```
import pypint
```

You are using Pint version 2018-03-22 and pypint 1.4.1

We first convert to GINsim model (multi-valued network) to Pint (automata network)

In [8]:

```
an = ginsim.to_pint(lrg)
```

In [9]:

```
smv_pint = pypint.to_nusmv(an)
smv_pint.add_ctls(properties)
smv_pint.alltrue()
```

Out[9]:

```
True
```
