# Supplementary material for: The CoLoMoTo Interactive Notebook: Accessible and Reproducible Computational Analyses for Qualitative Biological Networks
Source: Front Physiol. 2018 Jun 19;9:680. doi: 10.3389/fphys.2018.00680 (PMC6018415; doi:10.3389/fphys.2018.00680)
Supplement: Data Sheet 2 — The supplemental data “Notebooks” contains several short Jupyter notebooks which demonstrate different usage of the CoLoMoTo interactive notebook, listed in Table 2. The .ipynb files can be imported and executed within the Jupyter interface of the CoLoMoTo notebook, using the Docker image colomoto/colomoto-docker:2018-03-31. For each of these notebooks, a static HTML file previews the Jupyter rendering of the notebook, without any requirement. These notebooks can also be previewed and downloaded at https://nbviewer.jupyter.org/github/colomoto/colomoto-docker/tree/2018-03-31/tutorials. [file Data_Sheet_2.ZIP › Notebooks/demo-pint.html]

quick-tutorial


# Pint - Quick Tutorial¶

This document aims at giving a brief tutorial for Pint usage through its Python API.
A more complete tutorial can be found at http://loicpauleve.name/pint/doc, including installation instructions.

This tutorial can be followed either from the standard Python interpreter, or with IPython, and notably the Jupyter notebook web interface.

In the following, we assume that the `pypint` module has been loaded as follows:

In [1]:

```
import pypint
```

This notebook has been executed using the docker image `colomoto/colomoto-docker:2018-03-31`

You are using Pint version 2018-03-22 and pypint 1.4.1

When loaded with the Jupyter IPython interface, a menu provides shortcuts for the main Pint instructions.

## Model input¶

Pint relies on Automata networks framework which encompasses asynchronous Boolean and multi-valued networks.

The python module allows a seamless importation of models in different file formats. The conversion is made automatically and is ensured to be exact. The next sections show how to load a model, access and update its initial state, and saving and exporting to different formats. Finally, we show how to get basic informations on the automata network model.

### Loading a model¶

Pint supports multiple formats for specifying Boolean and multi-valued networks, in particular SBML-qual and GINsim. Most of the conversions are performed using BioLQM. See load() documentation for the full list of supported formats.

The loading of a model is done with the function `pypint.load` which returns an abstract object representing the automata network. The format is guessed from the file extension.
The argument can either be a local path to a file, or a URL which will be downloaded prior to loading:

In [2]:

```
m = pypint.load("http://ginsim.org/sites/default/files/drosophilaCellCycleVariants.zginml")
```

Downloading 'http://ginsim.org/sites/default/files/drosophilaCellCycleVariants.zginml'

Simplifying model...

6 state(s) have been registered: G0, Cycle\_Init, Endocycle\_init, Syncytial\_Cycles, NoCycD\_Endocycle, Ago\_Endocycle

The `load` function also supports importing models from CellCollective simply by supplying the URL to the model repository:

In [3]:

```
m2 = pypint.load("https://cellcollective.org/#2329/apoptosis-network")
```

Downloading 'http://api.cellcollective.org/model/export/2329?type=SBML'

Simplifying model...

Finally, within the Jupyter notebook web interface, a model can be uploaded by calling `load` without any argument. Once the file selected, it will be uploaded in the notebook, and loaded as a local file.

In [4]:

```
m3 = pypint.load()
```

### Initial state¶

An automata network comes with an initial state, associating to each automaton its starting local state.
Some formats, such as GINsim, allow to define different states that are identified with a name. In such a case, the `load` function displays the registered states.

The initial state of a model `m` is accessed using `m.initial_state`. It is a dict-like object which supports updating.

The recommended way to modify the initial state of the automata network is to use the `having` method to create a new copy of the model with supplied initial state modifications:

In [5]:

```
n = m.having(CycE=1)      # having can take keyword arguments
n = m.having({"Notch": 1, "Stg": 1})   # .. or a dictionnary
n = m.having("NoCycD_Endocycle")       # .. or the name of a registered state
```

See the Pint tutorial on model input for more functions and examples.

## Transient reachability analysis¶

The main features of pint relate to the transient reachability analysis, for which efficient over- and under-approximation have been designed.
Verifying reachability properties in automata networks is a PSPACE-complete problem, and is therefore hardly tractable for large systems.
Pint implements static analyses for the formal approximaton of reachability properties with a lower complexity. It consists in both over- and under-approximations, i.e., the verification of necessary *or* sufficient conditions.

The reachability properties are specfied in term of a *goal*. We first detail the different possible specifications for such a goal, and then illustrate various analyses of its reachability.

### Goal specification¶

In its simplest form, a goal is the local state of one automaton, for instance `g=1`. The goal is reached as soon as a state where `g=1` is reached. Note that there is no assumption on the stability of this state, this is why we refer to the *transient* reachability analysis.

Besides the single local state, a goal can be

- a (sub-)state, specified either as by a dict-like object or string representation of the (sub)-state
- a sequence of (sub-)state: for instance a `Goal("a=1","b=1")` is reached if one can reach a state where `a=1` and from which can be reached a state where `b=1`
- a disjunction of goals: `Goal("a=1")|Goal("c=1")` is reached if one can reach a state where either `a=1` or `c=1`.

In [6]:

```
from pypint import Goal # avoid typing pypint
simple = Goal("g=1")    # simple goal
substate = Goal("a=1,b=1")   # reach a state where both a=1 and b=1
seq = Goal("a=1,b=1", "c=1") # reach a state where a=1 and b=1 and from c=1 is reachable
alt = Goal("a=1", "b=1") | Goal("c=1") # either reach a state where a=1 and then a state where b=1, 
                                       # or reach a state where c=1
```

### Static reachability analysis¶

The reachability method takes as main argument the goal. It returns either `True` or `False` depending if the goal is reachable or not from the model initial state. By default, if the static analysis is not conclusive (i.e., the necessary condition is satisified, but the sufficient condition is not), pint falls back to exact model checking with ITS. This fall back can be deativated with the argument `fallback=None`. In this case, the method may also return `Inconc`.

In [7]:

```
invasion = pypint.load("http://ginsim.org/sites/default/files/SuppMat_Model_Master_Model.zginml")
```

Downloading 'http://ginsim.org/sites/default/files/SuppMat\_Model\_Master\_Model.zginml'

Simplifying model...

1 state(s) have been registered: initState\_1

In [8]:

```
invasion.having(DNAdamage=1,ECMicroenv=1).reachability("Apoptosis=1")
```

Out[8]:

```
True
```

### Identification of mutations to control goal reachability¶

One of the prime application of reachability analysis is the identification of perturbations which would control the goal reachability.

Pint offers an efficient static analysis which identify mutations for preventing a goal to occur. The analysis aims at being tractable on networks with hundreds of nodes.

The method oneshot\_mutations\_for\_cut takes as main argument the goal and returns a list of mutations which makes it impossible to reach.

In the following example, we compute the mutations which prevent both the activation of `Apoptosis` and `CellCycleArrest`. A mutation can be tested with the lock method which blocks the specfied automata to the given local states. It is guaranteed that the reachability returns false.

In [9]:

```
goal = Goal("Apoptosis=1") | Goal("CellCycleArrest=1")
invasion.initial_state["ECMicroenv"] = 1
invasion.initial_state["DNAdamage"] = 0
mutations = invasion.oneshot_mutations_for_cut(goal, maxsize=3, exclude={"ECMicroenv","DNAdamage"})
mutations
```

This computation is an *under-approximation*: returned mutations are all valid, but they may be non-minimal, and some solutions may be missed.

Limiting solutions to mutations of at most 3 automata. Use `maxsize` argument to change.

Out[9]:

```
[{'AKT1': 1},
 {'CDH1': 1, 'NICD': 0},
 {'CDH2': 1, 'NICD': 0},
 {'DKK1': 1, 'NICD': 0},
 {'CTNNB1': 0, 'NICD': 0},
 {'SNAI2': 1, 'ZEB2': 0, 'p21': 0},
 {'ZEB2': 0, 'p21': 0, 'p53': 0},
 {'AKT2': 1, 'ZEB2': 0, 'p21': 0},
 {'NICD': 0, 'SNAI2': 1, 'TWIST1': 0},
 {'NICD': 0, 'TWIST1': 0, 'p53': 0},
 {'ERK': 1, 'SNAI1': 1, 'ZEB2': 0},
 {'ERK': 1, 'SNAI2': 1, 'ZEB2': 0},
 {'ERK': 1, 'SNAI2': 1, 'p63': 1},
 {'ERK': 1, 'ZEB1': 1, 'ZEB2': 0},
 {'ERK': 1, 'ZEB1': 1, 'p63': 1},
 {'ERK': 1, 'ZEB1': 1, 'p53': 1},
 {'ERK': 1, 'ZEB2': 0, 'p53': 0},
 {'AKT2': 1, 'ERK': 1, 'ZEB2': 0},
 {'ERK': 1, 'miR200': 0, 'p63': 1},
 {'AKT2': 1, 'ERK': 1, 'p63': 1}]
```

In [10]:

```
invasion.lock({'DKK1': 1, 'NICD': 0}).reachability(goal)
```

Out[10]:

```
False
```

### Cut sets of paths to goal¶

Cut sets are sets of local states of automata which cover all the paths (or traces) from the initial state to any state where the goal is present. Therefore, disabling the local states of a cut set removes (cuts) all the paths to the goal, making it impossible to reach.

The method cutsets returns a list of cut sets of all the paths to the given goal.
It is tractable on very networks, up to several thousands of automata.

In [11]:

```
invasion.cutsets("Apoptosis=1",maxsize=3)
```

This computation is an *under-approximation*: returned cut-sets are all valid, but they may be non-minimal, and some cut-sets may be missed.

Limiting results to cut-sets with at most 3 elements. Use `maxsize` argument to change.

Out[11]:

```
[{'DNAdamage': 0},
 {'SNAI2': 0},
 {'ZEB2': 0},
 {'AKT1': 0},
 {'p63': 0},
 {'p53': 0},
 {'p53': 1},
 {'p73': 0},
 {'miR200': 0},
 {'miR34': 0},
 {'AKT2': 0},
 {'ERK': 0},
 {'CDH1': 0, 'ECMicroenv': 1},
 {'CDH2': 0, 'ECMicroenv': 1},
 {'DKK1': 0, 'ECMicroenv': 1},
 {'CTNNB1': 1, 'ECMicroenv': 1},
 {'CDH1': 0, 'CTNNB1': 0},
 {'CDH1': 0, 'NICD': 1},
 {'CDH2': 0, 'CTNNB1': 0},
 {'CDH2': 0, 'NICD': 1},
 {'CTNNB1': 0, 'DKK1': 0},
 {'DKK1': 0, 'NICD': 1},
 {'CTNNB1': [0, 1]},
 {'CTNNB1': 1, 'NICD': 1}]
```

In [12]:

```
invasion.disable(p53=1).reachability("Apoptosis=1")
```

Out[12]:

```
False
```

### Identification of bifurcation transitions¶

Given an initial state and a goal, a *bifurcation transitions* is a transition after which the goal is no longer reachable. Bifurcation transitions are local transitions of the automata network that are triggered in the transient dynamics. See [Fitime et al at WCB 2016] for more information.

Bifurcation transitions are helpful to understand differentiation processes, as they give the decisive modification which removed the capability to reach the goal.

The method bifurcations takes as first argument the goal for which the bifurcation transitions will be identified. The method may be tractable on networks with several hundreds of nodes. It also allows a `method="exact"` keyword for the complete identification of bifurcation transitions using model-checking. It is however not tractable on large networks.

The following computation can take a couple of minutes.

In [13]:

```
invasion.bifurcations("Apoptosis=1")
```

This computation is an *under-approximation*: returned transitions are all bifurcation transitions, but some may have been missed. Use `method="exact"` for complete identification.

Out[13]:

```
["AKT2" 0 -> 1" when "GF"=0 and "TGFbeta"=0 and "CDH2"=0 and "TWIST1"=1 and "NICD"=1 and "p53"=0 and "miR203"=0 and "miR34"=0",
 "AKT2" 0 -> 1" when "GF"=0 and "TGFbeta"=0 and "CDH2"=1 and "TWIST1"=1 and "p53"=0 and "miR203"=0 and "miR34"=0",
 "SNAI2" 0 -> 1" when "TWIST1"=1 and "p53"=0 and "miR200"=0 and "miR203"=0",
 "AKT2" 0 -> 1" when "GF"=1 and "TWIST1"=1 and "p53"=0 and "miR203"=0 and "miR34"=0",
 "SNAI2" 0 -> 1" when "TWIST1"=0 and "CTNNB1"=1 and "p53"=0 and "miR200"=0 and "miR203"=0",
 "SNAI2" 0 -> 1" when "TWIST1"=0 and "CTNNB1"=0 and "NICD"=1 and "p53"=0 and "miR200"=0 and "miR203"=0",
 "AKT2" 0 -> 1" when "GF"=0 and "TGFbeta"=1 and "TWIST1"=1 and "p53"=0 and "miR203"=0 and "miR34"=0"]
```

For each of the returned local transitions, there exists a state reachable from the initial state which (1) can reach the goal; and (2) after applying the bifurcation transition, the goal is no longer reachable.

### Goal-oriented model reduction¶

Pint can statically detect part of transitions that do not take part in minimal paths to the goal reachability. A path is minimal if and only it contains no cycle and all the transitions are causally related.

These transitions can then be removed from the model while preserving all the minimal paths to the goal and potentially reducing the reachable state graph significantly, and enhancing the tractability of model-checking.

In [14]:

```
len(invasion.local_transitions)
```

Out[14]:

```
244
```

In [15]:

```
red = invasion.reduce_for_goal("Apoptosis=1")
len(red.local_transitions)
```

Out[15]:

```
150
```

It is worth noting that the `reachability` method introduced above automatically performs the goal-oriented reduction prior the call to the model-checker.

See the Pint tutorial on transient reachability analysis for more functions and examples.

## State graph analysis¶

Besides static analyses of transient reachability, Pint offers standard state graph analysis.

### Fixpoints¶

Fixpoints are global states of the networks where no transition is possible.
Pint implements the complete fixpoints computation in automata networks as Boolean satisfaction constraints (SAT).
It should be tractable on networks with hundreds of components. Usually, the main limitation is the potentially very high number of fixpoints.

Let us illustrate the fixpoint computation on a medium size example:

In [16]:

```
th17 = pypint.load("http://ginsim.org/sites/default/files/Th_17.zginml")
```

Downloading 'http://ginsim.org/sites/default/files/Th\_17.zginml'

Simplifying model...

The method `fixpoints` returns the list of all the fixpoints of the network. The result is independent from the initial state. In the following example, we use Pandas to provide a pretty display of the returned list with Jupyter web interface. This is purely cosmetic.

In [17]:

```
fps = th17.fixpoints()
import pandas as pd # for pretty display of fixpoints
pd.DataFrame(fps)
```

Out[17]:

|  | GATA3 | IFNb | IFNbR | IFNg | IFNgR | IL12 | IL12R | IL18 | IL18R | IL4 | IL4R | IRAK | SOCS1 | STAT1 | STAT4 | STAT6 | Tbet |
| --- | --- | --- | --- | --- | --- | --- | --- | --- | --- | --- | --- | --- | --- | --- | --- | --- | --- |
| 0 | 0 | 0 | 0 | 0 | 0 | 0 | 0 | 0 | 0 | 0 | 0 | 0 | 0 | 0 | 0 | 0 | 0 |
| 1 | 1 | 0 | 0 | 0 | 0 | 0 | 0 | 0 | 0 | 1 | 1 | 0 | 0 | 0 | 0 | 1 | 0 |
| 2 | 0 | 0 | 0 | 1 | 1 | 0 | 0 | 0 | 0 | 0 | 0 | 0 | 1 | 1 | 0 | 0 | 1 |
| 3 | 0 | 0 | 0 | 2 | 1 | 0 | 0 | 0 | 0 | 0 | 0 | 0 | 1 | 1 | 0 | 0 | 2 |

### Reachable state graph¶

Pint also implements explicit reachable state graph analysis from the model initial state. Note that such a kind of approach is limited in scalability. On classical desktop computers, the state graph analysis can handle mostly up to a few (reachable) million states.

We illustrate on a small example the usage of the method `reachable_attractors` which returns the complete list of attractors reachable from the initial state. An attractor is described by a type (fixpoint or cyclic), a size, and an instance of state belonging to the attractor.

In [18]:

```
phage = pypint.load("http://ginsim.org/sites/default/files/phageLambda4.zginml",simplify=False)
```

Downloading 'http://ginsim.org/sites/default/files/phageLambda4.zginml'

3 state(s) have been registered: initState\_WT, initState\_Cro\_ect, initState\_CII\_ect

In [19]:

```
phage.initial_state
```

Out[19]:

```
{'CI': 0, 'CII': 0, 'Cro': 0, 'N': 0}
```

In [20]:

```
attractors = phage.reachable_attractors()
attractors
```

Out[20]:

```
[{'sample': {'CI': 0, 'CII': 0, 'Cro': 2, 'N': 0},
  'size': 2,
  'type': 'cyclic'},
 {'sample': {'CI': 2, 'CII': 0, 'Cro': 0, 'N': 0},
  'size': 1,
  'type': 'fixpoint'}]
```

The above result show that there are two attractors reachable from the initial state. One is a cyclic attractor which contains two states, and the other one is a fixpoint.

The following code use the sampled state of the cyclic attractor as initial state and compute the reachable state graph from it, showing the full composition of this cyclic attractor.

In [21]:

```
phage.having(attractors[0]["sample"]).reachable_stategraph() # display the cyclic attractor
```

```
# computing graph layout...
```

Out[21]:

xml version="1.0" encoding="UTF-8" standalone="no"?


SG


3

CI=0,Cro=3,CII=0,N=0

2

CI=0,Cro=2,CII=0,N=0

3->2


2->3

The initial state is represented with a gray background.

See the Pint tutorial on state graph analysis for more functions and examples.
