# Supplementary material for: The CoLoMoTo Interactive Notebook: Accessible and Reproducible Computational Analyses for Qualitative Biological Networks
Source: Front Physiol. 2018 Jun 19;9:680. doi: 10.3389/fphys.2018.00680 (PMC6018415; doi:10.3389/fphys.2018.00680)
Supplement: Data Sheet 2 — The supplemental data “Notebooks” contains several short Jupyter notebooks which demonstrate different usage of the CoLoMoTo interactive notebook, listed in Table 2. The .ipynb files can be imported and executed within the Jupyter interface of the CoLoMoTo notebook, using the Docker image colomoto/colomoto-docker:2018-03-31. For each of these notebooks, a static HTML file previews the Jupyter rendering of the notebook, without any requirement. These notebooks can also be previewed and downloaded at https://nbviewer.jupyter.org/github/colomoto/colomoto-docker/tree/2018-03-31/tutorials. [file Data_Sheet_2.ZIP › Notebooks/demo-interactive-fixpoints.html]

Fixpoints (interactive)


This notebook shows how to use `biolqm` to upload a model and compute its fixpoints.

In [ ]:

```
from colomoto_jupyter import tabulate
import biolqm
```

You can upload a model in any format supported by `bioLQM` (see https://github.com/colomoto/bioLQM#how-to-use-it), including GINsim (`.zginml` or `.ginml` files) and SBML-qual (`.sbml` files).

In [ ]:

```
lqm = biolqm.load("/tmp/colomoto5xd4zsjj_colomotoa6ug09_yphageLambda4.zginml")
```

The following cell code computes the fixpoints, and use `tabulate` to display the resulting table.

In [ ]:

```
fps = biolqm.fixpoints(lqm)
tabulate(fps)
```
