# Supplementary material for: The CoLoMoTo Interactive Notebook: Accessible and Reproducible Computational Analyses for Qualitative Biological Networks
Source: Front Physiol. 2018 Jun 19;9:680. doi: 10.3389/fphys.2018.00680 (PMC6018415; doi:10.3389/fphys.2018.00680)
Supplement: Data Sheet 2 — The supplemental data “Notebooks” contains several short Jupyter notebooks which demonstrate different usage of the CoLoMoTo interactive notebook, listed in Table 2. The .ipynb files can be imported and executed within the Jupyter interface of the CoLoMoTo notebook, using the Docker image colomoto/colomoto-docker:2018-03-31. For each of these notebooks, a static HTML file previews the Jupyter rendering of the notebook, without any requirement. These notebooks can also be previewed and downloaded at https://nbviewer.jupyter.org/github/colomoto/colomoto-docker/tree/2018-03-31/tutorials. [file Data_Sheet_2.ZIP › Notebooks/demo-cellcollective.html]

CellCollective - Knowledge Base


# CellCollective Knowledge Base¶

Models from CellCollective.org can be directly imported by CoLoMoTo tools (such as `bioLQM` or `Pint`) by giving their URL as the argument of the `.load` function, for instance, `lqm = biolqm.load("https://cellcollective.org/#2329/apoptosis-network")`.

In this notebook, we show how to use the API of the `cellcollective` python module to access metadata of network species, such as UnitProt and NCBI gene identifiers.

In [1]:

```
import cellcollective
```

This notebook has been executed using the docker image `colomoto/colomoto-docker:2018-03-31`

### Model loading from cellcollective.org¶

A CellCollective model can be imported using its URL on https://cellcollective.org.
Alternatively, you can also use "cellcollective://MODEL\_ID" syntax.

In [2]:

```
sbml = cellcollective.load("https://cellcollective.org/#2329/apoptosis-network")
```

Downloading 'http://api.cellcollective.org/model/export/2329?type=SBML'

The cellcollective python module supports basic access to the SBML-qual file, including the list of defined qualitative species:

In [3]:

```
sbml.species
```

Out[3]:

```
{'A20',
 'AKT',
 'APC',
 'Apaf1',
 'Apoptosis',
 'BAD',
 'BID',
 'BclX',
 'Cas12',
 'Cas3',
 'Cas6',
 'Cas7',
 'Cas8',
 'Cas9',
 'DNADamageEvent',
 'FADD',
 'GF',
 'GFR',
 'IAP',
 'IKK',
 'IkB',
 'JNK',
 'JNKK',
 'MEKK1',
 'Mdm2',
 'Mito',
 'NFkB',
 'NIK',
 'PI3K',
 'PIP2',
 'PIP3',
 'PTEN',
 'RIP',
 'TNF',
 'TNFR1',
 'TNFR2',
 'TRADD',
 'TRAF',
 'TRAF2',
 'cFLIP',
 'p53'}
```

### Access metadata¶

In most models on CellCollective, authors attached to nodes several metadata, including identifiers of biological species:

In [4]:

```
sbml.species_metadata("Cas3")
```

Out[4]:

```
{'GeneName': 'CASP3', 'NCBIGeneID': '836', 'UniProtID': 'P42574'}
```

The link to UnitProt knowledge or NCBI gene database can be obtained as follows:

In [5]:

```
sbml.species_uniprotkb("Cas3")
```

Out[5]:

https://www.uniprot.org/uniprot/P42574

In [6]:

```
sbml.species_ncbi_gene("Cas3")
```

Out[6]:

https://www.ncbi.nlm.nih.gov/gene/836

There exists several python interfaces to programmatically query information from these databases:

- using NBCI Gene ID: https://github.com/biocommons/eutils
- using UniProt ID: https://github.com/jdrudolph/uniprot

### Convert to bioLQM for model analysis¶

Finally, a CellCollective model can be imported in the `bioLQM` tool, for further processing, such as dynamical analysis and simulations.

In [7]:

```
lqm = cellcollective.to_biolqm(sbml)
```
